# Spatially Specific Liposomal Cancer Therapy Triggered by Clinical External Sources of Energy

**DOI:** 10.3390/pharmaceutics11030125

**Published:** 2019-03-16

**Authors:** Courtney van Ballegooie, Alice Man, Mi Win, Donald T. Yapp

**Affiliations:** 1 Experimental Therapeutics, BC Cancer, Vancouver, BC V5Z 1L3, Canada; cballegooie@bccrc.ca; 2 Faculty of Medicine, University of British Columbia, Vancouver, BC V6T 1Z3, Canada; 3 Faculty of Pharmaceutical Sciences, University of British Columbia, Vancouver, BC V6T 1Z3, Canada; aliceman321@gmail.com; 4 Department of Chemistry, Simon Fraser University, Burnaby, BC V5A 1S6, Canada; mi_mu_mu_win@sfu.ca

**Keywords:** triggered drug release, liposomes, ultrasound, magnetic fields, radiation

## Abstract

This review explores the use of energy sources, including ultrasound, magnetic fields, and external beam radiation, to trigger the delivery of drugs from liposomes in a tumor in a spatially-specific manner. Each section explores the mechanism(s) of drug release that can be achieved using liposomes in conjunction with the external trigger. Subsequently, the treatment’s formulation factors are discussed, highlighting the parameters of both the therapy and the medical device. Additionally, the pre-clinical and clinical trials of each triggered release method are explored. Lastly, the advantages and disadvantages, as well as the feasibility and future outlook of each triggered release method, are discussed.

## 1. Introduction

### Spatially Specific Liposomal Cancer Therapy Utilizing Medical Devices as Triggering Mechanism

There are many factors that contribute to the successful treatment of cancer and maximize tumor control. Surgery, chemotherapy, and radiotherapy are used in combination depending on tumor stage and grade. Molecular interrogation of the tumor highlights therapeutic targets specific to the patient’s tumor, and treatment options are optimized accordingly [1]. The importance of accurately imaging changes in the tumor volume and physiological functions has grown in tandem with the increasing use of targeted therapeutics [2]. Typically, the primary tumor is removed surgically, whenever possible, followed by adjuvant chemotherapy or radiation therapy. Chemotherapy is generally delivered systematically to kill cancer cells that may have migrated from the tumor, whereas radiation therapy is used locally to sterilize the surgical site. Unfortunately, each procedure carries its own adverse effects and risks; clinicians and patients must weigh the benefits against the risks before proceeding with a specific treatment regimen. In particular, using cytotoxic agents is associated with many unacceptable side-effects because these agents are potent cytotoxins that do not differentiate between normal and malignant cells. Unfortunately, despite efforts to mitigate the side effects, these negative effects can limit the drug dose that can be used with certain patients or reduce treatment compliance. Maximizing the anti-cancer activity of cytotoxic agents but minimizing their systemic toxicities, therefore, remains an important goal in optimizing chemotherapy treatments.

Liposomes have been particularly successful in modulating the biodistribution of cytotoxic drugs used in cancer treatment. In part, this is due to the versatility and classes of lipids that can be used to modify their distribution and release characteristics [3,4]. Liposomes are designed to encapsulate drugs, minimize drug release in circulation, accumulate at the tumor, and release drug locally when the bilayer is destabilized. This strategy enables drugs with proven activity to be preferentially delivered to the tumor site and reduce systemic side effects. However, the challenge is to balance drug encapsulation and release in a liposome so that the majority of the drug is released in the tumor, and not while in circulation.

More recently, there is growing interest in using external stimuli to trigger drug release from liposomes. The concept utilizes lipid carriers that are extremely stable and do not release significant amounts of drug at normal physiological conditions. However, the liposome would also be designed to be vulnerable to an external trigger that causes the liposome bilayer to become unstable and subsequently release its contents. Ideally, the stimuli would be focused on the tumor to ensure that only liposomes trapped there will release drug. Ultrasound (US) and magnetic fields (MF) used in magnetic resonance imaging (MRI) are primarily utilized for diagnostic purposes whereas radiation (RT) is used for imaging and treatment. These sources of external energies are of obvious interest for triggering drug release as they are already used clinically. The parameters for clinical imaging or therapy with US, MF, and RT are generally standardized whereas parameters used for drug release can vary greatly. Nonetheless, it is intriguing to speculate that clinically used parameters for US, MF, and RT could be modified to release drug from liposomes after imaging or concurrently during radiotherapy. Using imaging or therapeutic modalities to trigger intratumoral drug release on-command would help confine drug activity in the tumor and reduce systemic toxicities. However, clinical development of the strategies described could also open up novel treatments whereby, for example, imaging is used to confirm the presence of the drug carrier in the disease site before release. Furthermore, RT could be used to release drugs that potentiate the cell-killing effects of RT within the tumor. The latter strategy would facilitate drug-RT synergies that kill more cells than the sum of each separate approach. In this review, research work on the use US, MFs, and RT to trigger drug release from liposome drug carriers are summarized.

## 2. Ultrasound

### 2.1. Introduction

US triggered therapy relies on US waves compromising the integrity of a drug-loaded liposome to release its payload. By focusing the US transducer on the disease site, the emitted waves disrupt the bilayers of liposomes present in a defined area to release drug in a spatially-specific manner. The mechanisms causing drug release from the liposomes depends on the acoustic intensity, pulse frequency, pressure, duty cycle, and length of treatment. The effect of these parameters on the mechanism of release will also change depending on the tissue surrounding the drug carrier [5]. For example, tissues such as bone have a high absorption coefficient for acoustic waves and heat up more rapidly relative to other tissues with a low absorption coefficient [6,7]. Thus, a US treatment that is appropriate for soft tissue could potentially heat bone to lethal or damaging temperatures. In US-triggered drug release, acoustic parameters safe for the disease site must be matched to a liposome formulation that provides the desired mechanism of drug release. In the following section of the review, the mechanisms of drug release from liposomes using US and a summary of contributions from early pioneers in the field of US triggered release will be discussed.

### 2.2. Mechanism of Release

#### 2.2.1. Thermally Induced Release

As US waves are propagated through tissue, the acoustic wave can be reflected, transmitted, scattered, or absorbed. When absorbed, the acoustic wave energy is transformed into heat; however, to create localized heat within the area of interest, absorption of the acoustic energy must be greater than its diffusion [8]. In most cases, local heat can be generated using moderate intensities (several W/cm^2^), high duty cycles (up to 100%), moderate pressures (hundreds of kPa to MPa range), high frequencies (>0.5 MHz), and long treatment times (minutes to hours) [5]. Focused ultrasound (FUS) transducers are preferred because they generate heat more specifically, and at deeper tissue depths than transducers which emit planar, less-focused wave patterns. This helps mitigate heat generation in non-disease areas and reduce damage to normal tissues. It should be noted that high intensity frequency ultrasound (HIFU), in the context of drug delivery, can be undesirable as it has the potential to cause tissue ablation [5,9,10]. Thus, there is interest in using US with temperature sensitive liposomes (TSLs) that have a Tm higher than physiological temperatures for controlled drug release.

TSLs release their payloads at temperatures near or above their Tm [11]. This is due to fluidity changes within the liposomal membrane as it transitions from its gel (solid-like) phase to its liquid-crystalline phase. Within the gel phase, the permeability of the lipid bilayer is orders of magnitude less than that of the liquid-crystalline phase. The drug, therefore, remains within the liposome while it circulates throughout the body, and the liposome only releases the drug when surrounding temperatures rise to, or above, its Tm [12,13].

**Figure 1:**
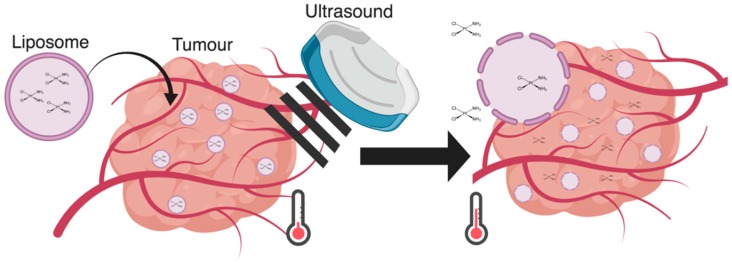
This figure depicts the accumulation of liposomes at the tumor site. Thermally induced release triggered by US then delivers the liposome’s drug payload.

Pioneers of TSLs, Yatvin et al., explored temperature-sensitive liposomes in the 1970s which were composed of 1,2-dipalmitoyl-*sn*-glycerophosphocholine (DPPC, Tm = 41.5 °C) and 1,2-distearoyl-*sn*-glycerophosphocholine (DSPC, Tm = 54.9 °C) for temperature-induced neomycin release in *E. coli*. Initially, work in temperature-sensitive liposomes displayed relatively slow drug release kinetics [11]. Despite this limitation, the DPPC DSPC TSL liposomes were tested in an in vivo subcutaneous Lewis lung tumor model where an implanted thermocouple maintained the temperature of the tumor at 42 °C. Although the authors were able to achieve a higher concentration of methotrexate (MTX) at the tumor site, confounding variables, such as increased blood flow and increased endothelial permeability, which occur due to continuous heating, made it unclear whether the increased MTX concentrations were due to the increased liposome accumulation at the site of the tumor or the increased liposomal drug release [14]. This study prompted further investigations of TSLs, such as improving the liposome’s drug release kinetics as well as identifying a method that could heat the tumor rapidly and locally. Since then, the original thermo-sensitive liposome formulations by Yatvin have been modified to improve drug release. This was mainly achieved through the alteration of the liposome’s lipid composition, such as including lysophospholipids, but the same has also been demonstrated through other methods, such as incorporating leucine-zippers to the membrane of the liposomes [12,15]. TSLs were later used with US induced heating to trigger drug release, as depicted in Figure 1, in the 1980s by Tracker and Anderson. It was then that the potential of using US to induce drug release in TSLs was first recognized due to the astounding 12-fold increase of drug accumulation at the site of interest under rapid, local US heating [16].

#### 2.2.2. Mechanically Induced Release

**Figure 2:**
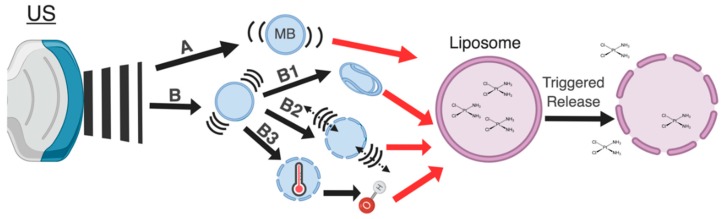
Schematic of the mechanisms associated with US that can be used to mechanically destabilize liposomes. The mechanisms are as follows: (**A**) US induced stable cavitation of a MB (**B**) US induced inertial cavitation of a MB; (**B1**) The production of a liquid microjets from a MB undergoing inertial cavitation; (**B2**) The production of shockwaves from a MB undergoing inertial cavitation; (**B3**) A MB collapsing and undergoing sonochemical changes.

Mechanical disruption of the liposome occurs via two mechanisms: stable cavitation coupled with radiation forces (RTFs) and inertial cavitation. Mechanical disruption of liposomes using US, however, requires the inclusion of compressible gaseous components, such as micelles, microbubbles (MBs), or liquid perfluorocarbon (PFC) droplets in the liposomal system. During cavitation, naturally nucleated or man-made gaseous materials contract and expand in response to the compression and refraction cycle of the acoustic wave. This, in turn, leads to a sustained oscillation of the bubble (stable cavitation) or to the rapid growth and ultimate collapse of the bubble (inertial cavitation). The type of cavitation that occurs depends highly upon the amplitude and frequency of the acoustic wave, as well as the size and material properties of the bubble [17,18,19]. The mechanical index (MI), the ratio of the in situ peak negative pressure (PnP) and the square root of the center frequency (F_c_), is a predictor of which process will dominate. Typically, an MI less than 0.8 MPa·MHz^−1/2^ results in stable cavitation while a higher MI leads to inertial cavitation [20]. Applications of cavitation not only include triggered release, as seen in Figure 2, but also include the modulation of blood perfusion, the permeabilization of the blood brain barrier (BBB), and even the breakdown of clots [21,22,23]. The modulation of perfusion and the permeabilization of the BBB allows for the modification of the nanoparticle’s biodistribution so that it may accumulate preferentially at the tumor and cross the BBB, respectively [21,24].

**Stable Cavitation and Radiation Forces**: During stable cavitation, a bubble expands and contracts about an equilibrium value; the oscillation about the bubble’s radius creates local swirling and fluid convection, termed micro-streaming, which induces shear stresses in the surrounding fluid [17]. This bubble-induced micro-streaming promotes the extravasation and delivery of circulating agents to target tissue [25]. Mechanistically, the shear stresses associated with US induced micro-streaming can rupture and deform liposomes or lyse the cell membrane (Figure 2B). These findings suggest that micro-streaming plays a pivotal role in drug delivery during instances of stable cavitation [26]. Micro-streaming caused by acoustic waves is a subset of forces, termed RTFs, that occur within the US field. These forces are able to displace particles and fluids not only via micro-streaming, but also through bulk streaming [25,27]. Bulk streaming occurs on a macro level where there is bulk, rather than local, fluid movement in the direction of the propagating acoustic wave. The RTFs generated via a stably cavitating bubble is highest at the driving frequencies near the microbubble’s resonance frequency [20,25,26]. This concept has driven the design of the liposome carriers to include bubbles with a larger radius that will generate greater acoustic forces and require a lower resonance frequency. These bubbles commonly range in size from 0.8–3 μm [28,29].

The most prominent drug delivery method, capitalizing on the effects of stable cavitation and RTFs, is lipid coated microbubbles, also known as gas filled lipospheres. One of the rationales for this construct was to increase the loading capacity of hydrophobic drugs despite the large size of the bubbles needed. This, in turn, resulted in the inner membrane of the liposome being replaced with a layer of oil so that hydrophobic small molecule agents could be dissolved and encapsulated at the interface between the bubble and lipid layer. This configuration of monolayer lipids is outside of the scope of this paper and reviews on this subject can be found elsewhere [30]. It should be noted, however, that many similar applications involving liposomes, such as tumor drug delivery and gene therapy, and targeted imaging are being pursued in this field [31,32,33,34,35,36].

**Inertial Cavitation**: Unlike stable cavitation where bubbles oscillate around an equilibrium point, bubbles undergoing inertial cavitation oscillate with increasingly large amplitudes. The radius of the bubble increases until it exceeds a critical limiting value, the bubble resonant radius (BRR), whereupon it collapses [18]. Inertial cavitation is facilitated by rectified diffusion, a process where liquid vapors diffuse into the bubble faster than they diffuse out of the bubble. The differential in incoming and outgoing vapors arises when the bubble’s radius and surface area increase due to the drop in internal pressure, which favors incoming over outgoing liquid vapors. Rectified diffusion is also affected by the change in concentration of the vapors in the bubble as the bubble oscillates due to the variation in the concentration gradient across the gas/liquid interface [18,19]. Factors that impact the resonant size of a bubble prior to collapse include the type of gas within the bubble, the surrounding medium, and the properties of the acoustic wave [18,37].

The three potential outcomes of inertial cavitation are (1) sonochemistry, (2) shockwaves, and (3) liquid microinjections [17,38] (Figure 2B). Sonochemistry is a sudden collapse of the bubble which generates momentarily high temperatures within the bubble’s core. These temperatures have been shown to reach 5000 K but last only microseconds due to rapid cooling rates (10^10^ K/s) [39,40,41]. A secondary effect of these high temperatures is to generate reactive oxygen (ROS) species in the surrounding area [42,43]. Sonochemistry, and specifically sonodynamic therapy, was first introduced by Umemura et al. in 1989 where the synergistic effect of hematoporphyrin and US were observed during the treatment of both in-vivo and in-vitro tumor models [44]. Extensive work has since been performed within this field that has subsequently identified the types and levels of ROS produced, and the impact of the MB concentration, US irradiation time, amplitude, and pressure on the production of the ROS species [38,43,45,46,47]. For example, an in vitro study by He et al. used the change in the absorption and fluorescence spectra of bovine serum albumin (BSA) in the presence and absence of ROS scavengers to indicate the extent of the ROS induced damage of the protein. In this study, it was demonstrated that higher bubble concentrations and longer treatment times led to greater protein damage [45]. Although these effects were studied using proteins, both lipid and membrane damage has also been reported in the literature [48,49,50]. One of the most pre-clinically relevant studies, which gives insights to the parameters that generate the greatest radical production in vivo, was performed by Prieur et al. in 2015. The lipid-radical byproducts of internal cavitation, malondialdehyde (MDA) and hydroxyterephthalic acid (HTA), were quantified using varying US fields in freshly excised pig tissue. Briefly, they found that cavitation related oxidative stress increases with an increasing amount of bubbles present, treatment exposure time, and peak negative pressure [51]. Additional studies focusing on mechanically triggered drug release from liposomes will be discussed in the formulation factors section.

Inertial cavitation is also able to generate shockwaves that can exceed amplitudes of 10,000 atmospheres depending on the size of the bubbles. These shock waves increase the permeability of membranous structures (sonoporation) [52]. This effect is twofold when using liposomes as it not only encourages the release of the therapeutic agent from the liposome but also increases the cell’s uptake of liposomes due to sonoporation [52,53]. Although the shock waves exist only for a short period of time, i.e., seconds to minutes, they have the ability to form large-spanning spatio-temporal pressure gradients [52]. Sonoporation has been demonstrated in vitro by Yudina et al. in 2010 where cell impermeable optical chromophores were added to a monolayer of C6 cells and were subjected to US. Only cells exposed to the US demonstrated fluorescent enhancement and this increased cell permeability phenomenon persisted even 24 h after exposure to US [54]. In vivo studies have further validated the presence of sonoporation, including delivering cell impermeable macromolecules (Bleomycin) to tumors, enhancing small molecule chemotherapy uptake in tumors, enhancing the blood brain barrier’s permeability to previously poorly permeable chemotherapies, and delivering genetic material, such as DNA plasmids, siRNA, and pDNA [55,56,57,58,59,60,61,62,63,64,65,66,67].

Liquid microjets form in non-uniform environments where bubbles collapse near a surface and produce high-velocity projections. The velocity of these microjets can reach hundreds of meters per second and deposit significant energy densities at the site of impact. In doing so, it is thought that the microjet can penetrate the tissue or generate secondary stress waves in the tissue [37,38,68,69]. One of the first ever recorded evidence for microjets via US induced cavitation was demonstrated and characterized by Bowden and Brunton in 1958 and 1961. In this pioneering work, they demonstrated that (1) the jet velocities of bubbles above a few hundred m/sec acted like solid projectiles, (2) damage to the surface of impact contained two parts, an irreversible and often erosive deformation and a secondary shearing and tearing of the surface with subsequent fracturing, and (3) the pressure generated at impact could be approximated with the liquid density, velocity of the acoustic wave, and jet velocity [70]. Since then, extensive mathematical and experimental modeling has been performed [71,72,73,74]. In particular, a pivotal paper was released in 1998 by Kodama and Takayama which not only elucidated how the characteristics of the bubble impacted the microjet produced but also how the microjets interact and influence excised tissues. Briefly, they identified that the initial radius of the bubble had a logarithmic correlation to the penetration depth and pit size (i.e., the size of the damaged area) achieved. They also demonstrated that the liquid jet penetration into the liver induces a shear force between the hepatocytes, thereby leading to the elongation and splitting of the nuclei [75]. In the context of cancer, liquid microjets and their secondary shockwaves have been shown to (1) induce permeability of the cell to enhance chemotherapy uptake, and (2) cause cell death, membrane damage, and alterations in cellular metabolism [76,77].

Although the three outcomes of inertial cavitation have been discussed separately, identifying the primary mechanism of drug release is often difficult and it is thought that the therapeutic outcome results from two or more of the possible consequences of inertial cavitation. For the purposes of this review, inertial cavitation will be considered as a single entity regardless of the multiple mechanisms at play.

### 2.3. Formulation Factors

#### 2.3.1. US Device Factors

As shown in Table 1, treatment parameters that can vary during treatment include (1) acoustic amplitude, (2) acoustic frequency, (3) duty factor, (4) pressure, and (5) treatment time. Often, varying one parameter will change the influence of another parameter on the type of triggered therapy achieved. An example of this was portrayed in Section 2.2 when describing the pressure and frequency parameters that would predict either stable or inertial cavitation. Table 1 also describes the common parameters used to achieve in vivo triggered release via the different mechanisms described in this review.

Examples of detailed in vivo parameters can be seen in Table 2. Experimental, in-vitro tests and mathematical modeling have been performed to determine how US parameters impact drug release from liposomes [80]. Cavitation induced release will be discussed first, followed by thermal triggered release. Briefly, Schroeder et al. demonstrated that clinically approved liposomes, including Doxil^®^, Stealth^TM^ Cisplatin, and methylpredinisolone hemisuccinate (MPS), when delivered under low frequency US (20 kHz), had a strong positive correlation of the % drug released with higher acoustic amplitudes (up to 7 W/cm^2^) and irradiation time (up to 180 s). The impact of the increasing amplitudes continued with no maximal value achieved, while the irradiation time began to level off after 120 s of exposure. Additionally, the duty cycle, whether it be pulsed (<100%) or continuous (100%), had no impact on the % of drug released. It should be noted, however, that these experiments were performed in a glass scintillation vial with an immersed US probe [81]. Due to this experimental setup, the pressure was not varied or made to mimic conditions of the body, such as that found in the capillary vessels. A later study by Afadzi tested similar parameters in an insonication chamber on liposomes composed of 52 mol% DEPC, 5 mol% DSPC, 8 mol% DSPE-PEG, and 35 mol% cholesterol. Similar to Schroeder, they identified a positive correlation using low frequency US (300 kHz) between the % drug released and higher acoustic frequencies, with maximal release at 10 W/m^2^, as well as a logarithmic correlation with exposure time. Contrary to Schroeder et al., however, they identified a positive correlation between the % drug released and the duty cycle (MI 2.4, 1.3 MPa, 180 s exposure, duty cycle ranged from 0–20%) [82]. This contrary finding could have been due to the % of duty cycles used. Further studies using a larger range of duty cycles should be performed.

Other US factors that can impact drug release include the (1) pulse duration (PD, i.e., the number of cycles multiplied by the inverse of the frequency), (2) pulse repetition frequency (PRF, i.e., the number of pulses per second), and (3) number of acoustic cycles (i.e., the number of acoustic oscillations per US pulse). Often, these parameters will not be specified, as they are related to the parameters in Table 1. For example, the duty factor and the PFR are directly related. Additionally, the PD is equivalent to the number of cycles multiplied by the inverse of the frequency. Therefore, because these factors are related to the initial five stated parameters, they will be covered only briefly in this review.

In the same study by Afadzi as described above, the % of drug release was also positively correlated with the PD and PRF [82]. A later study in 2016 by Lin et al. explored PD, PnP, and PRF in the context of the type and magnitude of cavitation induced. Here, they discovered that the onset of both stable and inertial cavitation exhibited a strong dependence on the PnP and PD and a relatively weak dependence on the PRF. Moreover, the amount of stable and inertial cavitation varied with the PRP. The amount of stable cavitation initially increased with increasing PnP until the pressure reached 0.5 MPa, where it rapidly decreased. By contrast, the amount of inertial cavitation recorded continuously increased with increasing PnP. Lastly, both PRF and PD positively correlated with both stable and inertial cavitation [83]. Another variable that was not previously discussed is the number of acoustic cycles applied. A paper by Mannaris and Averkiou identified the influence of the number of acoustic cycles applied on the microbubble by suspending the bubble in an enclosure that resembled capillaries. When applying the same acoustic parameters to a bubble (PRF = 100 Hz; *f* = 1 MHz) with an MI of 0.4, they found that increasing the number of cycles from 200 cycles to 1000 cycles had minimal effects on when the bubbles experienced inertial cavitation; this was likely caused by the high acoustic pressure used in the experiment. The authors speculate that the number of cycles could have a greater impact on the bubble’s oscillation when exposed to nondestructive pressures [84]. However, it should also be noted that specific parameters of the experiment, such as the presence and size of the bubble used, will also impact the parameters utilized with the US device [28,29].

When considering heat induced release, many of the US parameters are limited by physiological factors. For example, although higher temperatures can be achieved with greater pressures, such as between 2–3 MPa at 1 MHz, kidney and lung hemorrhaging begin to appear at 3–5 MPa and 2 MPa respectively and can, therefore, not be achieved safely in vivo [85,86,87]. Additionally, the treatment time is dependent on the biology of the tumor and can range drastically based on the volume and location of the tumor tissue. Small, superficial tumors will take a fraction of the time to heat (approximately 1 h) relative to deep lying larger tumors (can be more than 6 h based on size and location) [88]. The influence of the acoustic amplitude, also known as intensity, with respect to the heating of tissue was mathematically derived by Pierce in 1981. Put simply, the power deposited per unit volume of tissue was found to equal two times the local acoustic intensity multiplied by the absorption coefficient of the tissue [7]. Therefore, higher intensities would lead to a greater energy deposition, and thus, a greater generation of heat. Reviews that detail the mathematical modeling of heat transfer and heat deposition using US can be found elsewhere [20]. The high intensities necessary to heat tissues is reflected in the first two preclinical trials listed in Table 2. Lastly, the duty cycle will influence how often the tissue is exposed to these high intensity US waves. The higher the duty cycle, the more energy deposition there is with the highest being a continuous wave (100% duty cycle) [87]. The duty cycle used will often reflect the amount of heat necessary at the site of the tumor. For instance, using a continuous exposure can result in the thermal ablation of tissue (>60 °C) while a pulsed exposure can achieve mild hyperthermia (37–45 °C) [89]. Controlling the energy deposition in vivo, whether it be through the intensity of the US wave and/or the duty cycle used, is critical as vascular damage is suggested to appear at a local energy density of 0.3 mJ/mm^2^ [90].

#### 2.3.2. Liposomal Factors

The major liposomal factors that contribute to the liposome’s response to US include (1) the liposome’s composition, and (2) the physical state of the liposome’s bilayer. The liposome’s composition can be further broken down into three categories: (a) the presence of thermo-sensitive lipids, such as 1,2-dimyristoyl-*sn*-glycero-3-phosphocholine (DMPC) or 1-myristoyl-2-palmitoyl-*sn*-glycero-3-phosphocholine (MPPC), (b) the presence of surface-active molecules, such as detergents, and (c) the presence of cholesterol and polyethylene glycol (PEG). In a paper studying the impact of thermosensitive lipids on TSLs, Needham et al. identified that MPPC and DMPC lipid-containing liposomes, which lower the phase Tm, enabled enhanced drug release by local hyperthermia. The enhanced drug release at the liposome’s Tm was thought to occur due to the coexistence of the gel and liquid phase domains within the membrane. At the boundary regions between the two domains, a mismatch in molecular packing would occur, thereby facilitating the enhanced drug release. This phenomenon would be further enhanced by kinetically trapped MPPC lipids in the solid phase which, upon the gel-liquid crystalline phase transition, would leave the bilayer and enhance the permeability. In vitro findings by Needham et al. were later translated in vivo, and demonstrated significantly reduced tumor growth using Dox TSLs relative to the free Dox and a non-temperature sensitive Dox-containing liposome formulation [91]. Introducing structural irregularities within the membrane to disrupt the packing of the acyl chains is also the mechanism behind the increased drug release when introducing other unsaturated phospholipids. This hypothesis was tested by Huang and McDonald who showed that incorporating unsaturated diheptanoylphosphatidyl-choline (DHPC) into liposomes increased the release of encapsulated calcein upon US irradiation [92]. Surfactants, such as Triton and Tween, are also thought to destabilize the lipid bilayer. Indeed, a study involving two Triton and two Tween detergents showed a dramatically increased susceptibility of liposomes to US irradiation at concentrations that caused no observable increase in permeability in the absence of US [93]. Additionally, it was demonstrated that in cholesterol-free liposomes, Pluronic P105 sensitized liposomes to US irradiation when it was either in the presence of or directly incorporated with liposomes. The observed 10-fold increase in dye release, however, disappeared once cholesterol was incorporated into the lipid bilayer. This suggests that cholesterol has a protective effect between the interaction of Pluronic P105 and the lipid bilayer [94]. Interestingly, it was later demonstrated that increasing the cholesterol present in the lipid bilayer had a minimal but still statistically significant impact on dye release [95]. Lastly, the addition of PEG moieties will be discussed. While there are different methods of incorporating PEG into liposomal samples, such as the addition of PEG micelles or free PEG to the sample, PEGs covalently linked to phospholipids (PEG-lipids) and incorporated in the liposome’s bilayer will solely be discussed due to their clinical relevance. The effect of the PEG length and molar ratios of PEG-lipids was studied using low frequency US (LFUS) by Lin and Thomas. Briefly, they identified that the length of the PEG (PEG350-DPPE and PEG2000-DPPE) had no impact on the amount of dye released when using concentrations below a mole ratio of 0.1 PEG-lipid to PC were utilized [93,94]. The influence of the PEG length on liposomal release only occurred when high molar ratios of PEG-lipid, above 0.1 PEG-lipid to PC, were used. These studies demonstrated that a shorter PEG length at high molar ratios yielded higher levels of dye release than a longer PEG length at high molar ratios [93]. It was speculated that this phenomenon occurred in part due to the acoustic absorption of PEG moieties as well as the potential of shorter PEGs hindering the resolution of deformities in the liposomal bilayers [93,96]. More research is required to confirm the mechanism behind this observation.

It should be noted that the above studies utilized liposomes primarily comprised of 1,2-Distearoyl-*sn*-glycero-3-phosphoethanolamine (DSPE), DPPC, or egg phosphocholine (egg PC). Recent studies have explored replacing the major lipid constituent with dioleoylphosphatidylethanolamine (DOPE) in order to create a sonosensitive liposomes. A study by Evjen et al. demonstrated a 30% increase in Dox release using DOPE liposomes compared to liposomes comprising DSPE, and a 9-fold improvement in release extent when compared to l-α-phosphatidylcholine (HSPC) pegylated liposome when irradiating with US for 6 min at 40 kHz [97]. When investigating the interaction of the physical state of the liposome’s bilayer and US, Dunn and Tata and Maynard et al. identified enhanced US absorbance at the DMPC and DPPC liposome’s Tm. Briefly, they subjected liposomes comprised of DMPC or DPPC to 1.42 and 2.11 MHz US, respectively, and recorded the ultrasonic absorption and velocity of the samples. Enhanced ultrasonic absorbance only occurred at the phase Tm; below the phase transition, it was observed that US was hardly absorbed by the membrane [98,99]. These findings suggest that, when working at temperatures below the phase transition of the liposome, the mechanism of release is independent of the liposome’s absorbance of US but is dependent on the local cavitation and RTFs as well as heating of the surrounding tissue.

### 2.4. Future Perspectives

US is an emerging technology with the potential to be incorporated in the clinic for triggered delivery of liposomal drugs. As seen in Table 2, only the thermal release mechanism has proceeded to clinical trials at the time of writing of this review. This is in part due to the fact that US induced heating, such as HIFU, has already undergone multiple Phase II and III clinical trials and is currently in clinical practice in China [100]. Additionally, the safety of HIFU has been well documented experimentally in vivo and in patients. It was of initial concern that inertial cavitation and the shear forces produced using US would increase the cancer cell’s ability to dissociate from the primary tumor and form a metastatic site at a distal location. This, however, was found not to be the case as HIFU treatment did not increase the number of metastatic sites nor the number of circulating tumor cells [89,101]. In fact, HIFU has demonstrated such promise that it may one day serve as an alternative to the surgical resection of tumors. It has been well documented that when primary malignant tumors are surgically resected, their distal metastases begin to rapidly progress. Although there are many proposed mechanisms, such as the secretion of growth factors in response to the surgery or a shift in the pro- and anti-angiogenic factors secreted from the tumor itself, the best understood mechanism for this phenomenon thus far is the suppression of the immune system. Recent studies have suggested that HIFU can enhance cancer-specific immunity after treatment. Specifically, HIFU is thought to enhance the T cell-mediated immune response [102,103]. Currently, the prevailing two mechanisms are (1) that the ablated tumor tissue acts as an antigen source for the generation of antitumor immunity and (2) that HIFU enhances the release of heat-shock proteins which can then stimulate cytotoxic T-cells [103,104,105,106]. Thus far the benefits of HIFU have been described but an important clinical consideration is the safety and side effect profile of the treatment. The most frequently occurring adverse events are moderate pain, with approximately <15% of patients experiencing this symptom, followed by transient fever and skin toxicities [107,108,109,110]. Interestingly, the Phase I clinical trials using mild hyperthermia for liposomal drug release (TARDOX and DIGNITY) reported either no adverse events or a low prevalence of grade 3–4 adverse events respectively. [111,112,113] This was likely due to the parameters used as the recorded level of tumor heating was found to be 40 °C rather than the 60 °C needed for tissue ablation [112,113]. Another advantage of US that was not discussed previously, but that is prevalent in these clinical trials, is the ability of the US treatments to be administered in a single treatment session rather than in multiple sessions as seen in radiotherapy. [111,112,113] While US does have obvious advantages as a treatment method, such as being minimally invasive and displaying a low adverse effect profile, there are some limitations to the technology. Specifically, there are three major disadvantages of US as a treatment method. The first is the inability of US to penetrate air-filled viscera. This will limit the ability of US to be utilized with tumors located in areas such as the lungs, intestines, or bladder. The second disadvantage also involves the tumor’s location, particularly if there is no acoustic window for the US to reach the tissue of interest. For example, if there is a structure obscuring the tumor with a high absorption coefficient, such as bone, the acoustic wave may be unable to reach its intended target. The third major disadvantage to US is the long treatment times discussed in Section 2.3.1 [88]. Despite these limitations, the results of the clinical trials thus far and the benefits seen in HIFU treatment highlight the potential of combining liposomal triggered release, in conjunction with either mild or moderate hyperthermia, as a promising option for cancer treatment. While mechanically triggered release has shown promising results preclinically, more research is required to better evaluate this mechanism of release as an alternative option for cancer treatment.

## 3. Magnetism for Triggered Drug Release

### 3.1. Introduction

MFs are an attractive way to release drugs from liposomes due to the technique’s non-invasiveness, absence of ionizing radiation, and physiologically benign field frequencies and amplitudes [123]. In the clinical management of cancer, MFs are used primarily with contrast agents to diagnose and stage tumors. The technique is commonly used in cases where the tumor is made of softer tissue, as MRI scans provide images that enhance soft tissue contrast [124]. Aside from MRI, one of the best-known uses of magnetism in cancer is alternating magnetic field (AMF) induced hyperthermia. AMFs are characterized by rapid and regular changes in the MF’s direction. Radiofrequency (low frequency AMFs) can penetrate deep into the body unhindered. However, high frequency, high amplitude AMFs will induce electric currents in tissue and can raise bulk tissue temperatures to lethal limits because of the tissue’s resistance to electrical currents. If lethal temperatures are reached (>42 °C), the heat-induced damage to cancer cells cannot be repaired and the cells die [125]. Unfortunately, the effects of AMFs are not specific to malignant tissue and the surrounding normal tissue in the field may also be damaged, thereby limiting the maximum frequency and amplitude of AMFs that can be safely used in humans. Currently, there is no consensus on the safety limits for AMFs, but AMFs of 100 kHz and amplitude <10 kA/m have been used safely in clinical trials [126,127]. Additionally, the penetration depth and safety profile of low energy AMFs are advantageous when compared to other external triggers for activating nanomaterials, such as light or X-rays, which are limited by their shallow penetration depths and ionizing damage to normal tissue, respectively. [128].

**Figure 3:**
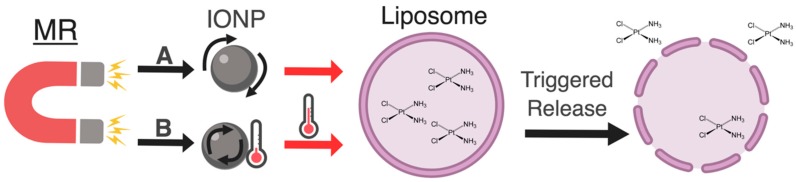
Schematic of the mechanisms associated with MR that can be used to destabilize liposomes. The mechanisms are: (**A**) MR induced mechanical disruption; (**B**) MR induced hyperthermia.

The heat generated by magnetic nanoparticles (MNPs) upon application of AMFs has been used to raise the bulk temperatures of malignant tissue to lethal limits in a process called magnetic hyperthermia [129]. The bulk heating of tissue with magnetic hyperthermia has also been used to release drug from nano-composites, liposomes, polymers lipid structures, or cyclodextrin conjugated to MNPs [130,131,132,133,134,135,136,137]. In the following section of the review, the use of MFs to disrupt liposomes associated with MNPs, by either inducing heat or by mechanical motion to cause the release of their payload, will be discussed (Figure 3).

### 3.2. Delivery Using Heating and Mechanical Motion

In the field of temperature induced drug delivery, liposomes have been used to deliver drugs to tumors by taking advantage of the slightly elevated temperatures in malignant tissue. With a Tm only a few degrees above physiological temperatures, these liposomes were able to release their encapsulated drugs within the malignant tissue [138]. However, due to the liposome’s Tm, these TSLs did not prevent drug loss as the liposomes circulated throughout the body. To circumvent the issue of nonspecific drug release, liposomes with Tms significantly higher than normal tissue temperatures were subsequently used in conjunction with tissue heating. Drug release would, therefore, not be triggered by normal body temperatures until they reached the disease site where temperatures were increased past the liposome’s Tm with induced hyperthermia. Tissue hyperthermia can be induced in various ways, including the use of US (as seen previously in Section 2.2.1) and, in some cases, magnetism—particularly MFs interacting with liposomes and magnetic particles (MPs) [139,140,141].

Using MNPs in the tumor to potentiate the tissue heating effects of AMFs can raise tissue temperatures at lower magnetic frequencies and amplitudes that spare normal tissue from damage [127]. The incorporation of MNPs within the liposome itself has the potential to limit AMF-induced heating to within the drug carrier so that bulk heating of the tissue is unnecessary. A study demonstrated selective hypothermia in vivo and in vitro using magnetoliposomes under a low-frequency AMF to promote lipid membrane permeability from local heating. However, the relatively high heating of the composite particles led to concerns of overheating normal body tissues [142]. In order to overcome injury to healthy tissues caused by the overheating of the particles, other groups embedded the MNPs within the drug carrier to minimize heating of the tissue directly for their drug release studies. For example, Amstad et al. encapsulated iron oxide particles (IOPs) within liposomes and succeeded in controlling the timing of release by increasing the permeability of the liposomes without destroying them [143]. They concluded that AMF-induced heat was confined to the liposomes and subsequently spared normal tissue. Similarly, another study triggered the release of carboxyfluorescein from TSLs containing IOPs through AMF induced local heating [144].

Disrupting a liposome’s lipid bilayer by local heating using encapsulated MNPs (magnetic fluid hyperthermia) to release drugs is common, but efforts to mechanically disrupt the bilayer with MFs have also been made. Most work investigating drug release by mechanical means have been done with MNPs located within the lipid bilayer. In an article studying the toxicity effects on cells, Kim et al. used microdiscs that oscillated when an alternating magnetic force is applied. Cell membrane integrity was compromised partly due to the oscillating microdiscs attached to the cell surface; therefore, it is not inconceivable that liposomal membranes containing MPs under the field would also be disrupted [145]. Drug release has also been observed from magnetoliposomes due to the mechanical vibrations of the IOPs [146]. Furthermore, the rotation of IOPs, induced through a dynamic magnetic field (DMF), can injure cell membranes. In contrast to using AMFs, DMF uses lower frequency parameters that cause unique rotations of individual particles around their own axes. This produces rotational shear forces that lower membrane integrity, without thermal effects [147]. Mechanical disruption of the membrane can also be achieved with pulses of an MF, as opposed to an alternating one. In this study, the authors showed drug release from liposomes after treatment with short magnetic pulses that disrupted the lipid bilayer. They further concluded that drug release from mechanical disruption of the liposomes was less harmful to the drug payload as an increase in temperature could potentially damage the drug [148].

### 3.3. Mechanisms of Release

The inherent magnetism of MNPs distinguishes them from other types of particles. MNPs behave as a single magnetic moment with an absolute value several orders of magnitude higher than that of single atoms, and can be remotely actuated or detected by an MF [149]. When MNPs are exposed to AMFs, their magnetic moments move to align with the field direction, but ‘relax’ or rotate back to their original alignment when the field is removed. The realignment or ‘relaxation’ of the magnetic moment represents a net energy loss that is released as heat. The MNP can physically rotate in the tissue and release heat into the surrounding tissue (Brownian relaxation), or the MNP remains stationary while its internal magnetic moment rotates with the field and releases heat at the surface of the MNP (Néel relaxation) [150]. Under the action of AMFs, this process happens many times, causing significant increases in temperature [151]. The amount of heat released by MNPs depends on the core magnetic material, the hydrodynamic diameter and shape of the particle, and the frequency and amplitude of the AMFs [152]. Brownian relaxation forms the basis for heating up bulk tissue in magnetic hyperthermia and the subsequent release of drug from TSLs. In contrast, Néel relaxation is thought to have less impact on the surrounding tissue; reports indicate that temperatures >40 °C have been estimated at MNP surfaces when subjected to AMFs, but that the temperature gradients between MNPs and their surroundings fall off very steeply [153,154]. The use of magnetic nanoparticles that undergo Néel relaxation for local heating of the lipid bilayer with AMFs benign to normal tissue would alleviate unintended heating of normal tissue.

MNPs can also behave as nanomagnets that align themselves to the plane of an MF, and this alignment can also be used to kill cancer cells. When the field is rotated or changes its direction, the particles move along with the field. Studies in which the particles are attached to cell membranes indicate that the particles can create mechanical forces strong enough to rupture the cell and cause cell death [155,156]. 

### 3.4. Formulation Factors

MNP induced liposomal drug release relies on both the properties of the MNP as well as the liposome. These components, therefore, allow for a range of conditions that can be tuned to change the release characteristics of the delivery system. The three main parameters that can be modified for drug release include (1) the MF’s frequency and amplitude, (2) the composition of the lipid bilayer, and (3) the properties of the MNP such as the shape, size and composition. The tunability of these parameters are important and must be considered when developing delivery systems that are biocompatible and only release drug at the disease site in response to a magnetic field.

The properties of the MF are important when considering the thermal or mechanical disruption of the liposome for drug release [148,157]. The frequency of the MF is perhaps the factor most commonly changed, and a range of AMF frequencies have been used in studies for drug release, with higher frequencies leading to the higher motion of the nanoparticles as well as local heat production. This is also true of the strength of the field [158]. MFs can generate enough heat to irreversibly damage tumor cells yet may also damage healthy tissue at very high strengths. The suggested safe range for strength and frequency is up to 37 kA/m and 500 kHz [159]. Alternatively, pulses of strong MFs can be used to disrupt the lipid bilayer by using the motion of the nanoparticles, as opposed to generated heat [148].

Lipid bilayers used in drug delivery vary greatly in composition, due to the vast selection of fatty acids available for liposome production. To ensure that the liposomes destabilize at the proper temperature and release their drug payload, it is important to choose bilayer components carefully. Pradhan et al. used a liposomal formulation of DPPC:cholesterol:DSPE-PEG2000:DSPE-PEG2000-Folate at an 80:20:4.5:0.5 molar ratio in a delivery system containing MNPs. Their experiment demonstrated a significant release of the drug payload when exposed to an MF that increase the temperature a few degrees above ambient body temperature [160]. Peller et al. also used a DPPC, DSPC, and 1,2-dipalmitoyl-*sn*-glycero-3-phosphodiglycerol (DPPG2) liposome (Tm~43 °C) at a molar ratio of 50:20:30, respectively, for their TSLs in a magnetic hyperthermia study observing drug release using MRI markers. Here, they were able to successfully target drug delivery using temperature control [161].

MNPs can have an infinite number of formulations, with options including, but not limited to, their size, shape and composition. Generally, their size ranges from 1 to 100 nm in diameter [162]. These particles are commonly made from multiple elements including iron, cobalt, nickel and platinum. MPs are mainly classified based on their structure between magnetic alloy particles and magnetic metal oxide particles, the latter of which is used in drug delivery [163]. Metal oxides, particularly iron oxides, have already been seen as a promising candidate in magnetic hyperthermia, demonstrating abilities to kill cells locally through magnetically induced heating [129]. The properties of the IOPs impact the efficiency of these particles to confer heating to their immediate environments. For example, there is an increase in the specific absorption rate (SAR), the rate at which radiofrequency energy is absorbed, when nanorods are used compared to spherical and cubic forms due to their 1-dimensional nature. SAR is an important aspect to triggering the release of drugs as a higher absorption rate equates to particles heating up more as a certain amount of energy is applied. Therefore, lower energies and fewer particles are needed within the tissues for drug release. Das et al. found that the SAR can be changed by adjusting the aspect ratio of nanorods; higher aspect ratio of nanoparticles resulted in higher SAR values [164]. Furthermore, the ellipsoidal shape of magnetic nanorods can influence two effects when in an AMF; extra heat is released compared to nanospheres due to shape anisotropy and the nanorods dynamically reorient to the field [165]. These two properties are important as the former equates to extra heat release efficiency meaning fewer particles and lower field intensities are required during hyperthermia treatments, while the latter effect could be used to develop nanorobots in magnetic hyperthermia through controlled motion and orientation. The hyperthermic efficiency of nanorods, relative to their cubic and spherical counterparts of similar magnetic volumes, was further confirmed elsewhere. One study also compared the heating efficiency of nanospheres versus deformed cubes (orthopods) ranging from 17–47 nm. Throughout this size range, orthopods had a higher heating capacity and changing the size and shape of these particles changes the SAR [166]. Additonally, iron oxide nano-octopods were found to have better heating efficiency as compared to spheres [167]. Another factor that can impact the SAR of the NPs is their composition. A 2018 paper by Espinosa et al. compared the SAR values of maghemite-based IONPs (Fe_3_O_4_) and cobalt ferrite NPs (CoFe_2_O_4_) at clinically relevant settings (470 kHz) and found a small, but statistically significant, increase in SAR using CoFe_2_O_4_ NPs [168]. While other compositions have been studied, including MnFe_2_O_4_ and NiFe_2_O_4_, another factor that has been suggested to impact the IONP’s SAR value is the iron oxide’s oxidation state [169]. Overall, these experiments demonstrate that the heating efficiencies of MNPs can be modulated by the MNP’s shape, size, and composition.

### 3.5. Future Perspectives

Using MFs as a release trigger is relatively new. The use of AMFs and IOPs encapsulated within liposomes has not yet been examined in clinical trials. Using MNPs in conjunction with thermosensitive liposomes for triggered release is a promising modality for cancer treatment. Some challenges, however, remain in the clinical application of these systems. One of the most pressing is determining the optimal MF parameters that maximize targeted heating of tissue or particles without damage to healthy cells. In this case, using MNPs to potentiate the heating effects of the MF are an advantage. Increasing MF strength may increase the SAR or heating potential of injected MPs, but too high of a field strength would lead to non-specific heating of the tissues [126]. Because of this, there has been much interest in producing MNPs with superior SAR values [164].

It is important to understand the toxicity, biocompatibility, and biodistribution of MNPs when using them in a triggering system. The biocompatibility of MNPs is linked to both the immune system response following its administration and to the intrinsic toxicity of the MNP and/or of its biodegradation metabolites. Factors that can influence the MNP’s toxicity include their surface coating, size, and surface charge. Typically, toxicities can be avoided using compatible coatings (such as PEG and starches), small sizes (within the nanoparticle range) with appropriate doses, and nearly neutral charges (+/−10 mV). The chemical composition of MNPs also plays a role in their toxicity. IOPs, for example, have been found to be safe at high doses (100 s to 1000 s of mg/kg) via oral, intravenous, intraperitoneal, and subcutaneous administration. Once IOPs are injected, the IOPs are exposed to opsonization and accumulate in macrophages of the reticuloendothelial system. This includes organs such as the liver, spleen, and bone marrow. Despite this accumulation, major toxic side effects are rare as cells are able to incorporate the iron from the IOPs into their endogenous iron metabolism. If an iron overload does occur, however, the tissues may experience oxidative stress and injury to their cell membrane [149]. Cellular iron overload is rare but can be overcome by changing the biodistribution of IOPs through their magnetic properties. This allows the MNPs, via an external MF, to be guided to the site of interest [170,171,172,173]. Although MNPs may accumulate in tissues that are not of interest in the absence of guiding MFs, there seems to be promise in the sequestering capabilities of the particles in the context of cancer. One study done on fibrosarcoma tumor bearing mice looked at a novel formulation of co-encapsulated La_0.75_Sr_0.25_MnO_3_ and IOPs that, under hyperthermia, resulted in tumor reduction by up to 3.6 fold, with little to no drainage of the particles to other organs in the body [140]. Future prospects of utilizing MNPs in conjunction with liposomes is promising as both components have been clinically approved as single agents (such as Doxil^®^, ThermoDox^®^, Caelyx^®^ Feraheme^®^, Feridex^®^, Gastromark^®^, etc.). There is potential for developing MPs as agents that react to hyperthermia and release liposome encapsulated drugs on-demand. At present, however, most studies are pre-clinical and much work remains to identify specific applications that take advantage of their unique, synergistic properties for clinical use.

## 4. X-ray Radiation

### 4.1. Introduction

Radiation therapy is one of the most effective modes of cancer treatment given the recent advancements in defining the spatial precision and depth penetration of ionizing radiation. More than 50% of all cancer patients receive radiotherapy over the course of their treatment with a curative or palliative intent [174]. By irradiating tumors with high energy photons or ion beams, cancer cells and the surrounding vasculature are irreparably damaged leading to tumor death [175,176]. Although radiotherapy is non-specific and can damage healthy tissue along the path of the photons, it remains the major course of treatment for primary non-metastasized solid tumors [174,177]. Concurrent RT and chemotherapy are also used in cancer treatment, particularly for unresectable tumors. Clinically, head and neck cancer patients with unresectable disease are treated with concurrent RT and cisplatin (CPT) to take advantage of drug-X-ray synergies for tumor control [178,179]. Unfortunately, in many patients, the systemic toxicity of CPT (hearing loss, kidney and nerve damage) is dose limiting [179,180]. Thus, using a delivery system where CPT is released locally at the irradiated site by X-rays would (1) minimize systemic chemotoxicity; (2) potentiate the efficacy of X-rays, and (3) potentially control the tumor with lower doses of radiation, chemotherapy, or both. Liposomes have demonstrated utility in chemotherapy as drug delivery vehicles by prolonging circulation time and increasing drug retention in tumors with several formulations used clinically [181]. Thus, it is not inconceivable that radiation-sensitive liposomes could be incorporated into pre-existing treatment plans for concurrent use with traditional radiotherapy. In comparison to other spatially-specific release systems, X-ray-triggered liposomal drug release is a relatively new concept. However, based on recent research focused on radiosensitization with gold nanoparticles, there is strong evidence suggesting that more efficient and effective systems can be designed to use radiation as a modality for triggered drug release [182].

**Figure 4:**
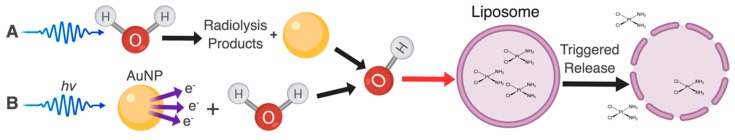
Schematic of the mechanisms associated with RT that can be used to destabilize liposomes. The mechanisms are as follows: (**A**) The interaction of radiation with water to produce radiolysis products that can interact with AuNPs to amplify hydroxy radical production. These radicals can then destabilize liposome bilayers for triggered drug release; (**B**) The interaction of radiation with AuNPs to produce secondary electrons (such as Compton scattering and Auger electrons) which can then interact with water to produce hydroxy radicals. These radicals can then destabilize liposome bilayers for triggered drug release.

The mechanism responsible for inducing the destabilization of the liposomal membrane is the radiosensitization effect [183,184], Figure 4. Radiosensitizers, such as gold nanoparticles, enhance the local radiation dose through the increased absorption of low and medium-energy X-rays and subsequent ejection of reactive secondary electrons [185]. A study by Sicard-Roselli et al. describes the direct and indirect mechanisms by which hydroxyl radicals are produced from gold nanoparticles irradiated in water. The direct mechanism produces hydroxyl radicals through the emission of electrons or lower energy photons from gold nanoparticles which interact with water, while the indirect mechanism involves the interaction of radiolysis products with gold nanoparticles which then eject electrons that interact with water [184]. The hydroxyl radicals produced through both pathways react with nucleic acids, proteins, and lipids located within their vicinity. In particular, reactive oxygen species are known to simultaneously: (i) initiate lipid peroxidation, a process which entails the abstraction of hydrogen atoms from lipid fatty acid chains, (ii) form peroxyl radicals, and (iii) convert fatty acid side chains into lipid hydroperoxides [183]. Although there have been no direct studies examining the mechanisms of radiosensitization in conjunction with liposomes, in theory, the local production of hydroxyl radicals and secondary electrons mediated by embedded radiosensitizers should cause lipid peroxidation and liposomal bilayer destabilization when irradiated, thereby triggering drug release [183,184].

To date, there have been several hundred studies exploring the potential therapeutic use of gold nanoparticles for radiosensitization [186]. The effect was initially shown by Hainfeld et al. who found a four-fold increase (86% versus 20%) in one-year survival rates for mice receiving both gold nanoparticles and X-ray therapy versus X-ray therapy alone. The mice were injected with 1.9 nm diameter gold nanoparticles (up to 2.7 g of Au/kg body mass) and irradiated with a 250 kVp X-ray beam [187]. In 2005, a Monte Carlo study, based on the aforementioned mouse study, was published by Cho, estimating a physical dose enhancement factor (DEF) of at least two-fold [188]. More than thirty reports have demonstrated radiosensitization effects in vitro with DEFs generally ranging from 1.1 to 1.9, while more than ten reports have shown radiosensitization effects in animal studies, showing that treatment with gold nanoparticles and X-ray cause tumor regression, or increased cell kill [189]. The initial understanding of the mechanism behind radiosensitization was attributed to physical factors, such as the high atomic number and photoelectric cross section of gold [190]. Monte Carlo simulations were used to discern the type and number of electrons emitted depending on variables such as the source, type, and energy of X-rays, as well as the size, concentration, and coating of gold nanoparticles. Generally, it was found that small gold nanoparticles at a high concentration, when irradiated with keV photon beams, generate the highest DEFs. However, as the in vivo and in vitro radiosensitization effects were often greater than predicted DEF values, it has now become well understood that complex chemical and biological interactions, such as the generation of hydroxyl radicals as mentioned above, are also involved radiosensitization, and therefore, require further investigation [189].

### 4.2. Formulation Factors

#### 4.2.1. Radiation Type and Energy

Physical dose enhancement depends largely upon the energy and type of the incoming radiation. Kilovoltage (KV) photons with energies above the k-edge of gold (80.7 keV) have been shown to produce maximal dose enhancement due to the ability of these photons to excite the lowest-lying K-shell electrons. These electrons are ejected by the photoelectric effect and cause the subsequent emission of lower energy secondary electrons from the gold atom, known as the Auger cascade. Although a majority of the electrons are reabsorbed by other atoms in the gold nanoparticle, 1 to 7 electrons from each gold atom ultimately escape to interact with the environment [191]. Leung et al. reported that these electrons could travel 3 µm to 1 mm from the gold nanoparticle, while Jones et al. found that the dose enhancement effect was significant only a few microns away [192,193]. Photons with energies under the k-edge of gold (for example, 40–50 keV) have also been shown to produce radiosensitization effects through the ejection of higher shell (L, M, N) electrons and a localized Auger cascade, as the mass energy absorption coefficient of gold is over 100 times greater than soft tissue in the 40 to 50 keV energy range. However, due to the poor penetration of lower energy X-ray beams through soft tissue, this strategy would not be clinically feasible unless brachytherapy seeds were implanted in close proximity to the nanoparticles [190].

Although higher dose enhancement factors were observed using kilovoltage photon beams, megavoltage (MV) photons have also been shown to produce gold nanoparticle-mediated radiosensitization [194,195]. For 4 and 6 MV photon beams, dose enhancement factors ranging from 1.01 to 1.07 were predicted by Cho, and therefore, were not initially considered for dose enhancement [188]. However, in an in vivo mouse study, Chang et al. demonstrated that 25 Gy of 6 MeV radiation could produce significant tumor volume reduction in the presence of gold nanoparticles [196]. This could be explained by the increased absorption of secondary species produced by the ionization of water [177,190]. More recently, Yang et al. showed that the incorporation of gold nanoparticles into a chemoradiation regiment using cisplatin and 2 Gy of 6 MV radiation caused a 19% decrease in cell survival compared to cisplatin and radiation therapy alone [197]. Again, the observed dose enhancement was greater than predicted by Monte Carlo stimulations, indicating that physics dosimetry plays a smaller role in MV radiosensitization [191]. As MV photon beams are often used for radiotherapy and have a greater depth penetration in tissue, they are important for clinical applications of radiosensitization [198].

Gold nanoparticles have also been shown to interact with ion beams which is being explored for treatment because of its specificity in dose deposition attributed to its defined Bragg peak [199]. In a theoretical study, Verkhovtsev et al. showed that ion beams caused the collective electronic excitation of the surface plasmon in metal nanoparticles [200]. This effect was found to be particularly strong for noble metals due to the high excitability of the surface plasmon where the relaxation energy released causes the subsequent ejection of reactive electrons [177,200].

#### 4.2.2. Gold Nanoparticle Size and Concentration

The size and concentration of the nanoparticles affect the degree of radiosensitization, since a greater number of gold atoms being irradiated generally causes greater dose enhancement [186]. Thus, higher concentrations of gold nanoparticles of the same size or larger gold nanoparticles at the same concentration produce greater dose enhancement effects [190,191]. However, when considering the optimal size of gold nanoparticles for a given mass of gold, smaller nanoparticle clusters produce greater dose enhancement effects. As the diameter of the gold nanoparticles increases, more of the secondary electrons and radiation are absorbed by the nanoparticle core, thereby reducing the energy available to interact with the surrounding environment [191]. In a Monte Carlo study, Lechtman et al. (2011) showed that a greater number of low energy Auger electrons were released by smaller nanoparticles while a greater number of high energy photoelectrons were released by larger nanoparticles [191]. This size effect has also been shown in a simulated cell study where 2 nm gold nanoparticles produced greater cell deaths than 50 nm gold nanoparticles when irradiated [201].

### 4.3. Future Perspectives

Currently, studies on X-ray triggered liposomal drug release are very limited. To our knowledge, only one study exists—that by Deng et al.—in which the authors used X-rays to trigger release from liposomes in vitro and in vivo. The system consisted of liposomes embedded with gold nanoparticles and verteporfin, a photosensitizer, where 19% of encapsulated calcein was released upon irradiation with 4 Gy of 6 MeV photons. Increased gene silencing and cell death were observed in vitro with the triggered release of antisense oligonucleotides and chemotherapy drugs, respectively. In a xenograft mouse model, X-ray triggered liposomes were shown to produce a 74% reduction in colorectal tumor volume compared to the control with phosphate buffered saline [182]. In a different study by Lukianova-Hleb et al., the authors relied on the increased endosomal uptake of AuNPs and liposomes in cancer cells to colocalize the NPs for triggered release. This paper demonstrated the synergy between liposomes, AuNPs, low-energy short laser pulses, and X-rays to induce plasmonic nanobubbles and ROS formation for the destabilization of the liposomes. Despite the absence of AuNP conjugation or encapsulation within the liposome, this paper alludes to the possibility of relying on the tumor’s biology to bring a carrier and triggering component in close enough proximity to each other to allow for triggered release [202]. Although these studies showed that X-rays are a promising modality for X-ray triggered liposomal drug release, many areas of possible development remain to increase the amount of drug released. For example, the amount of drug released could be improved using liposomal formulations containing higher concentrations of embedded gold nanoparticles [190]. Additionally, protein nanoparticles, such as Albumin, could possibly be used in place of liposomes since hydroxyl radicals interact strongly with proteins as well [203].

As X-ray-triggered drug release is still in its early stages of development, a better understanding of gold nanoparticle embedded liposomes, their toxicities, and the effects of radiotherapy fractionation is needed before clinical translation can be considered. Formulation factors affecting radiosensitization using gold nanoparticles have been well studied, but not in the context of liposomes. Since the incorporation of more gold nanoparticles increases local dose enhancement but destabilizes the membrane at high concentrations, an optimization of the two factors is needed [190,204]. To date, there has been one study outlining the in vitro pharmacokinetics of gold nanoparticle embedded liposomes. The intravenous injection of gold nanoparticle embedded liposomes (100 to 120 nm) into a fibrosarcoma mouse model resulted in the accumulation of gold nanoparticles in the liver, spleen, kidney, and intestines, but none in the tumor site [205]. This could possibly be due to the specific formulation of the liposome, as liposomes with diameters ranging from 100 to 300 nm have typically been found to accumulate near tumors due to the enhanced permeability and retention (EPR) effect [206,207]. Additionally, actively targeting the tumor using targeting ligands could increase the uptake efficiency of NPs in cancer cells. Significant progress has been made in this area of research both at the preclinical and at the clinical level and can be found in detail elsewhere [208]. A better understanding of the long-term toxicology of gold nanoparticles is also necessary, especially at the clinical level. Since surface chemistry, routes of administration, and dosages used vary extensively across pre-clinical toxicology studies, different results are found in the literature for nanoparticles of a given size [209]. For example, one study showed that gold nanoparticles ranging from 8–37 nm caused hepatocellular toxicities, while another showed that 13 nm PEG-capped gold nanoparticles caused no systemic toxicities [210,211]. Although the FDA has not approved any gold nanoparticle-based drugs for clinical use, a clinical trial of Aurimune^®^, which carries tumor necrosis factor into tumors, has successfully passed its first phase [212,213].

As radiotherapy can cause damage to normal tissue surrounding the tumor, careful consideration must be taken to limit the dose of radiation delivered during RT [177]. Therefore, the use of liposomes in conjunction with gold nanoparticles that promote radiosensitization is an attractive triggered therapy approach when taking into account the negative side effects that come from high dose RT. A feasible strategy for clinical translation would be to incorporate X-ray triggered drug release into existing treatment plans which use concurrent radiotherapy with chemotherapy. Examples include head and neck cancers, upper esophagus cancers, small cell lung cancer, and cervical cancer [179,214,215,216,217]. In particular, head and neck cancers and small cell lung cancer treatment plans recommend the use of over 60 Gy of radiation with concomitant chemotherapy where the total dose is fractionated to 2 Gy daily [217]. To maximize X-ray triggered drug release, irradiation should occur when the greatest concentration of drug loaded liposomes are found in the tumor site. Additionally, larger doses of radiation could be incorporated at this point to increase drug release if the treatment allows. Lastly, synergistically radiation-activated drugs, such as those used for photodynamic therapy, could be encapsulated and used for deep lying tumors that near infrared or visible light would be unable to reach [218]. Future prospects in this field are promising and experimental simulation of a clinical dosing schedule should be explored to better characterize the X- ray triggered liposomal release system as a whole.

## 5. Conclusions

**Figure 5:**
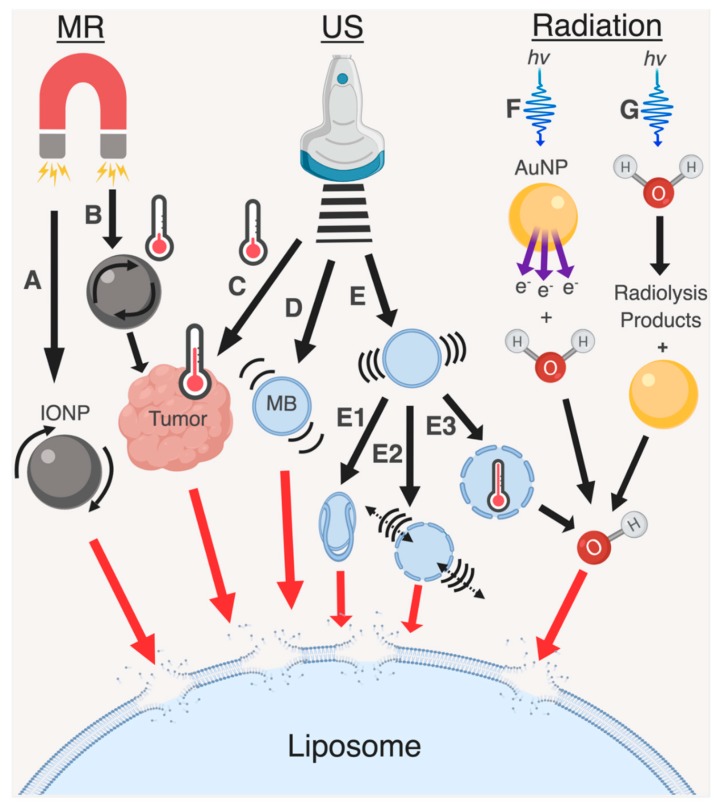
Schematic of the mechanisms associated with MR, US, and RT that can be used to destabilize liposomes. The mechanisms are as follows: (**A**) MR induced mechanical disruption; (**B**) MR induced hyperthermia; (**C**) US induced hyperthermia; (**D**) US induced stable cavitation of a MB; (**E**) US induced inertial cavitation of a MB; (**E1**) The production of a liquid microjets from a MB undergoing inertial cavitation; (**E2**) The production of shockwaves from a MB undergoing inertial cavitation; (**E3**) A MB collapsing and undergoing sonochemical changes; (**F**,**G**) The interaction of radiation with AuNPs and water to produce hydroxyl radicals that destabilize liposome bilayers.

This review explores the use of energy sources, including US, MFs, and external beam radiation, to trigger the delivery of drugs from liposomes in a tumor in a spatially-specific manner. The mechanism(s) of drug release that can be achieved using liposomes in conjunction with the external trigger were investigated for each of the energy sources. Figure 5 summarizes the mechanisms that can be achieved in each modality and the commonalities, such as hyperthermia and hydroxyl radical formation, found between some of the devices. While this paper identified the growing interest and advantages in using external stimuli to trigger drug release from liposomes, it also demonstrated the drawbacks associated with each method. Themes such as the range of penetration depth and off-target tissue damage, or lack thereof, were discussed in the advantages and disadvantages for each of the energy sources. Considerations such as these must be taken into account on a case by case basis and will impact the types of cancers that can be targeted with each modality. Furthermore, this review also detailed the treatment’s formulation factors and explored the parameters of both the therapy and the energy source. Each energy source identified a correlation between the size and concentration of their corresponding mutually exclusive particle (such as the MB’s, IONPs, or AuNPs) with the treatment’s experimental impact. Understanding each method’s formulation factors will aid in the development of future therapies which are susceptible to influence from external stimuli. Additionally, the pre-clinical and clinical trials of each triggered release method were explored. At the time of writing this review, only US used in conjunction with liposomes, specifically HIFU induced liposomal release, had clinical trials in motion as a method for cancer therapy. While these represent only three clinical trials, this only further highlights the feasibility and positive future outlook of utilizing the energy sources found in medical devices as external stimuli to induce liposomal release in the context of cancer therapy.

## Figures and Tables

**Table 1:** Common parameters used to induce thermal, stable cavitation, or inertial cavitation in vivo using US.

Treatment Parameters	Thermal	Stable Cavitation	Inertial Cavitation
Acoustic Amplitude	High to Moderate (several W/cm^2^) [5,78]	Low to Moderate (a few hundred mW/cm^2^ or less) [78]	Low to Moderate (a few hundred mW/cm^2^ or less) [78]
Acoustic Frequency	Moderate frequencies (0.5–1.5 MHz) [5]	Low to Moderate Frequencies (1 MHz or less) [51,78]	Low to Moderate Frequencies (1 MHz or less) [51,78]
Duty Factor	High duty cycles (up to 100%) [5]	Low duty cycles (as low as 1%) [51,78]	Low duty cycles (as low as 1%) [51,78]
Pressure	Moderate Pressure (100’s of kPa to MPa) [5]	Low Pressure (below 500 kPa) [79]	Moderate Pressure (above 500 kPa) [79]
Treatment Time	Long treatment times (minutes to hours) [5]	Short treatment times (a few minutes or below) [51]	Short treatment times (a few minutes or below) [51]

**Table 2:** Summary of preclinical and clinical cancer treatments for US induced therapy release using liposomes. (Prf = pulse repetition frequency, TAT = total acoustic power, Statistically significant = *, cw = continuous wave, amp = amplitude, *f* = frequency, ns = not specified).

Delivery System	Release Type	Animal/Tumor Model	Dosing	Parameters: *f*, Duration, Amp, Pulse *f*	Outcome	Ref.
ThermoDox^®^	Thermal	Murine mammary adenocarcinoma; BALB/c	2 mg/kg single injection	Prf of 1 Hz for a total of 1 MHz; 15–20 min; 1300 W/cm^2^; 120 pulses 10% duty cycle	* Tumor volume reduction	[114]
Prohance^®^ & dox-loaded TTSL	Thermal	Rat subcutaneous 9 L gliosarcoma; 344	5 mg/kg single injection	1.4 MHz; 2 × 15 min; 117 W/cm^2^; cw	* Dox accumulation in the tumor	[115]
Prohance^®^ & dox-loaded iLTSL	Thermal	Rabbit/VX2 tumor	5 mg/kg single injection	1.2 MHz; 4 × 10 min; ns; ns	ns	[116]
ThermoDox^®^	Thermal	Rabbit/VX2 tumor	5 mg/kg single injection	ns; 3 × 10 min; ns; ns MR-HIFU clinical system, parameters ns	* Dox accumulation in the tumor	[117]
Stealth^TM^ cisplatin	Mechanical	Murine lymphoma (J6456); BALB/c	15 mg/kg single injection	20 kHz; 120 s; 5.9 W/cm^2^; cw	* Tumor volume reduction	[118]
DVDMS liposomes conjugated to MBs	Mechanical	Murine breast cancer (4T1); BALB/c	4.0, 2.0, or 0.4 μg/single injection	1.0 MHz; 3 min; TAT 3 W; 30% duty cycle	* Tumor volume reduction	[119]
Caelyx^®^	Mechanical	Murine prostate cancer (CWR22); BALB/c	3.5 mg/kg single injection	40 kHz; 4 min; 12 W/cm^2^; ns	Tumor volume reduction	[120]
DEPC-based Dox-loaded liposomes	Mechanical	Murine prostate adenocarcinoma (PC-3); BALB/c	Not specified	Prf of 250 Hz for a total of 300 kHz or 1 MHz; 10 min; ns; 5% duty cycle	* Dox accumulation in the tumor	[53]
Doxil^®^	Mechanical and permeabilization	Rat 9 L gliosarcoma; Sprague-Dawley	5.67 mg/kg single injection	Prf of 1 Hz for a total of 1.7 MHz; pressure 1.2 MPa, burst length: 10 ms, duration: 60–120 s	* Tumor regression and long-term survival	[21]
Doxil^®^	Mechanical and permeabilization	Rat 9 L gliosarcoma; Sprague-Dawley	5.67 mg/kg single injection	690 kHz; pressures amp 0.55–0.81 MPa; burst length: 10 ms; prf: 1 Hz; duration: 60	* Tumor regression and long-term survival	[121]
ThermoDox^®^	Thermal	Phase I DIGNITY Clinical Trial; Breast Cancer	20 mg/m^2^–50 mg/m^2^, up to 6 doses, 21 days apart	ns; 1 h; ns; ns	Safe to move onto Phase II Clinical Trial	[111]
ThermoDox^®^	Thermal	Phase II DIGNITY Clinical Trial; Breast Cancer	40 mg/m^2^–50 mg/m^2^, up to 6 doses, 21 days apart	ns; 1 h; ns; ns	Expansion of Phase II Clinical Trial	[122]
ThermoDox^®^	Thermal	Phase I TARDOX Clinical Trial; Liver Metastases	50 mg/m^2^, up to 6 doses, 21 days apart	0.96 MHz; 33.2–80.0 min	Safe to move onto Phase II Clinical Trial	[112,113]

## Abbreviations

1,2-dimyristoyl-*sn*-glycero-3-phosphocholine	(DMPC)
1,2-dipalmitoyl-*sn*-glycero-3-phosphodiglycerol	(DPPG2)
1,2-dipalmitoyl-*sn*-glycerophosphocholine	(DPPC)
1,2-distearoyl-*sn*-glycerophosphocholine	(DSPC)
1-myristoyl-2-palmitoyl-*sn*-glycero-3-phosphocholine	(MPPC)
Alternating magnetic field	(AMF)
Amplitude	(Amp)
Blood brain barrier	(BBB)
Bovine serum albumin	(BSA)
Bubble resonant radius	(BRR)
Center frequency	(F_c_)
Cisplatin	(CPT)
Continuous wave	(CW)
Diheptanoylphosphatidyl-choline	(DHPC)
Dioleoylphosphatidylethanolamine	(DOPE)
Distearoyl-*sn*-glycero-3-phosphoethanolamine	(DSPE)
Dose enhancement factor	(DEF)
Dynamic magnetic field	(DMF)
Enhanced permeability and retention	(EPR)
Egg phosphocholine	(egg PC)
Focused ultrasound	(FUS)
Frequency	(*f*)
High Intensity Focused Ultrasound	(HIFU)
Hydroxyterephthalic acid	(HTA)
Iron oxide particles	(IOPs)
Kilovoltage	(KV)
l-α-phosphatidylcholine	(HSPC)
Low frequency US	(LFUS)
Magnetic fields	(MF)
Magnetic nanoparticles	(MNPs)
Magnetic particles	(MPs)
Magnetic resonance imaging	(MRI)
Malondialdehyde	(MDA)
Mechanical index	(MI)
Megavoltage	(MV)
Methotrexate	(MTX)
Methylpredinisolone hemisuccinate	(MPS)
Microbubbles	(MBs)
Not specified	(NS)
Peak negative pressure	(PnP)
Perfluorocarbon	(PFC)
Polyethylene glycol	(PEG)
Pulse duration	(PD)
Pulse repetition frequency	(PFR)
Radiation	(RT)
Radiation forces	(RTFs)
Reactive oxygen	(ROS)
Specific absorption rate	(SAR)
Temperature sensitive liposomes	(TSLs)
Total acoustic power	(TAT)
Ultrasound	(US)

## References

[1] Tsimberidou A.-M. (2015). Targeted Therapy in Cancer. Cancer Chemother. Pharmacol..

[2] Gerwing M., Herrmann K., Helfen A., Schliemann C., Berdel W.E., Eisenblätter M., Wildgruber M. (2019). The Beginning of the End for Conventional RECIST—Novel Therapies Require Novel Imaging Approaches. Nat. Rev. Clin. Oncol..

[3] Zylberberg C., Matosevic S. (2016). Pharmaceutical Liposomal Drug Delivery: A Review of New Delivery Systems and a Look at the Regulatory Landscape. Drug Deliv..

[4] Allen T.M., Cullis P.R. (2013). Liposomal Drug Delivery Systems: From Concept to Clinical Applications. Adv. Drug Deliv. Rev..

[5] Mitragotri S. (2005). Healing Sound the Use of Ultrasound in Drug Delivery and Other Therapeutic Applications. Nat. Rev. Drug Discov..

[6] Goss S.A., Johnston R.L., Dunn F. (1980). Compilation of Empirical Ultrasonic Properties of Mammalian Tissues. II. J. Acoust. Soc. Am..

[7] Pierce A.D. (1981). Acoustics: An Introduction to Its Physical Principles and Applications. Phys. Today.

[8] Fry W.J., Wulff V.J., Tucker D., Fry F.J. (1950). Physical Factors Involved in Ultrasonically Induced Changes in Living Systems: I. Identification of Non-Temperature Effects. J. Acoust. Soc. Am..

[9] Halliwell M.A. (2010). Tutorial on Ultrasonic Physics and Imaging Techniques. Proc. Inst. Mech. Eng. Part H J. Eng. Med..

[10] Bailey M.R., Khokhlova V.A., Sapozhnikov O.A., Kargl S.G., Crum L.A. (2003). Physical Mechanisms of the Therapeutic Effect of Ultrasound (a Review). Acoust. Phys..

[11] Yatvin M.B., Weinstein J.N., Dennis W.H., Blumenthal R. (1978). Design of Liposomes for Enhanced Local Release of Drugs by Hyperthermia. Science.

[12] Mabrey S., Sturtevant J.M. (1976). Investigation of Phase Transitions of Lipids and Lipid Mixtures by Sensitivity Differential Scanning Calorimetry. Proc. Natl. Acad. Sci. USA.

[13] Ruocco M.J., Siminovitch D.J., Griffin R.G. (1985). Comparative Study of the Gel Phases of Ether- and Ester-Linked Phosphatidylcholines. Biochemistry.

[14] Weinstein J.N., Magin R.L., Yatvin M.B., Zaharko D.S. (1979). Liposomes and Local Hyperthermia: Selective Delivery. Science.

[15] Al-Ahmady Z.S., Al-Jamal W.T., Bossche J.V., Bui T.T., Drake A.F., Mason A.J., Kostarelos K. (2012). Lipid-Peptide Vesicle Nanoscale Hybrids for Triggered Drug Release by Mild Hyperthermia in Vitro and in Vivo. ACS Nano.

[16] Tacker J.R., Anderson R.U. (1982). Delivery of Antitumor Drug to Bladder Cancer by Use of Phase Transition Liposomes and Hyperthermia. J. Urol..

[17] Margulis M.A. (1993). Sonochemistry and Cavitation.

[18] Young F.R. (1989). Cavitation.

[19] Crum L.A. (1984). Acoustic Cavitation Series: Part Five Rectified Diffusion. Ultrasonics.

[20] Coussios C.C., Roy R.A. (2008). Applications of Acoustics and Cavitation to Noninvasive Therapy and Drug Delivery. Annu. Rev. Fluid Mech..

[21] Treat L.H., McDannold N., Zhang Y., Vykhodtseva N., Hynynen K. (2012). Improved Anti-Tumor Effect of Liposomal Doxorubicin after Targeted Blood-Brain Barrier Disruption by MRI-Guided Focused Ultrasound in Rat Glioma. Ultrasound Med. Biol..

[22] Ammi A.Y., Linder J.R., Zhao Y., Porter T., Siegel R., Kaul S. (2015). Efficacy and spatial distribution of ultrasound-mediated clot lysis in the absence of thrombolytics. Thromb. Haemost..

[23] Belcik J.T., Davidson B.P., Xie A., Wu M.D., Yadava M., Qi Y., Liang S., Chon C.R., Ammi A.Y., Field J. (2017). Augmentation of Muscle Blood Flow by Ultrasound Cavitation is Mediated by Atp and Purinergic Signaling. Circulation.

[24] Burke C.W., Alexander I.V., Timbie K., Kilbanov A.L., Price R.J. (2014). Ultrasound-activated Agents Comprised of 5FU-bearing Nanoparticles Bonded to Microbubbles Inhibit Solid Tumor Growth and Improve Survival. Mol. Ther..

[25] Doinikov A.A., Bouakaz A. (2010). Acoustic Microstreaming around an Encapsulated Particle. J. Acoust. Soc. Am..

[26] Marmottant P.S. (2003). Controlled Vesicle Deformation and Lysis by Single Oscillating Bubbles. Nature.

[27] Shi X., Martin R.W., Vaezy S., Crum L.A. (2002). Quantitative Investigation of Acoustic Streaming in Blood. J. Acoust. Soc. Am..

[28] Zhao S., Borden M., Bloch S.H., Kruse D., Ferrara K.W., Dayton P.A. (2004). Radiation-Force Assisted Targeting Facilitates Ultrasonic Molecular Imaging. Mol. Imaging.

[29] Zhang Y., Guo X., Zhang D., Gong X. Evaluation of the Effects of Secondary Radiation Force on Aggregation of Ultrasound Contrast Agents. Proceedings of the 20th International Congress on Accoustics.

[30] Unger E.C., Porter T., Culp W., Labell R., Matsunaga T., Zutshi R. (2004). Therapeutic Applications of Lipid-Coated Microbubbles. Adv. Drug Deliv. Rev..

[31] Unger E.C., McCreery T.P., Sweitzer R.H., Caldwell V.E., Wu Y. (1998). Acoustically Active Lipospheres Containing Paclitaxel: A New Therapeutic Ultrasound Contrast Agent. Investig. Radiol..

[32] Suzuki R., Namai E., Oda Y., Nishiie N., Otake S., Koshima R., Hirata K., Taira Y., Utoguchi N., Negishi Y. (2010). Cancer Gene Therapy by IL-12 Gene Delivery Using Liposomal Bubbles and Tumoral Ultrasound Exposure. J. Control. Release.

[33] Shortencarier M.J., Dayton P.A., Bloch S.H., Schumann P.A., Matsunaga T.O., Ferrara K.W. (2004). A Method for Radiation-Force Localized Drug Delivery Using Gas-Filled Lipospheres. IEEE Trans. Ultrason. Ferroelectr. Freq. Control.

[34] Yang C.Y., Liu H.W., Tsai Y.C., Tseng J.Y., Liang S.C., Chen C.Y., Lian W.N., Wei M.C., Lu M., Lu R.H. (2015). Interleukin-4 Receptor-Targeted Liposomal Doxorubicin as a Model for Enhancing Cellular Uptake and Antitumor Efficacy in Murine Colorectal Cancer. Cancer Biol. Ther..

[35] Wang C.-H., Huang Y.-F., Yeh C.-K. (2011). Aptamer-Conjugated Nanobubbles for Targeted Ultrasound Molecular Imaging. Langmuir.

[36] Unger E.C., Mccreery T.P., Shen D., Wu G., Sweitzer R., Wu Q. (1998). Gas-Filled Liposomes as Ultrasound Contrast Agents for Blood Pool, Thrombus-Specific and Therapeutic Applications. J. Acoust. Soc. Am..

[37] Suslick K.S., Nyborg W.L. (1990). ULTRASOUND: Its Chemical, Physical and Biological Effects. J. Acoust. Soc. Am..

[38] Suslick K.S. (1988). Ultrasound. Its Chemical, Physical, and Biological Effects.

[39] Suslick K.S., Meyers R.A. (2001). Sonoluminescence and Sonochemistry. Encyclopedia of Physical Science and Technology.

[40] McNamara W.B., Didenko Y.T., Suslick K.S. (1999). Sonoluminescence Temperatures during Multi-Bubble Cavitation. Nature.

[41] Didenko Y.T., McNamara W.B., Suslick K.S. (1999). Temperature of Multibubble Sonoluminescence in Water. J. Phys. Chem. A.

[42] Weissler A. (1959). Formation of Hydrogen Peroxide by Ultrasonic Waves: Free Radicals. J. Am. Chem. Soc..

[43] Riesz P., Christman C.L. (1986). Sonochemical Free Radical Formation in Aqueous Solutions. Fed. Proc..

[44] Umemura S., Yumita N., Nishigaki R., Umemura K. Sonochemical Activation of Hematoporphyrin—A Potential Modality for Cancer Treatment. Proceedings of the IEEE Ultrasonics Symposium.

[45] He L.L., Wang X., Wu X.X., Wang Y.X., Kong Y.M., Wang X., Liu B.M., Liu B. (2015). Protein Damage and Reactive Oxygen Species Generation Induced by the Synergistic Effects of Ultrasound and Methylene Blue. Spectrochim. Acta Part A Mol. Biomol. Spectrosc..

[46] Kondo T., Mišík V., Riesz P. (1996). Sonochemistry of Cytochrome C. Evidence for Superoxide Formation by Ultrasound in Argon-Saturated Aqueous Solution. Ultrason. Sonochem..

[47] Miller D.L., Thomas R.M., Buschbom R.L. (1995). Comet Assay Reveals DNA Strand Breaks Induced by Ultrasonic Cavitation in Vitro. Ultrasound Med. Biol..

[48] Leung K.S., Chen X., Zhong W., Yu A.C.H., Lee C.-Y. (2014). Microbubble-Mediated Sonoporation Amplified Lipid Peroxidation of Jurkat Cells. Chem. Phys. Lipids.

[49] Rahman M.M., Ninomiya K., Ogino C., Shimizu N. (2010). Ultrasound-Induced Membrane Lipid Peroxidation and Cell Damage of Escherichia Coli in the Presence of Non-Woven TiO_2_ Fabrics. Ultrason. Sonochem..

[50] Tsuru H., Shibaguchi H., Kuroki M., Yamashita Y., Kuroki M. (2012). Tumor Growth Inhibition by Sonodynamic Therapy Using a Novel Sonosensitizer. Free Radic. Biol. Med..

[51] Prieur F., Pialoux V., Mestas J.-L., Mury P., Skinner S., Lafon C. (2015). Evaluation of Inertial Cavitation Activity in Tissue through Measurement of Oxidative Stress. Ultrason. Sonochem..

[52] Pecha R., Gompf B. (2000). Microimplosions: Cavitation Collapse and Shock Wave Emission on a Nanosecond Time Scale. Phys. Rev. Lett..

[53] Eggen S., Afadzi M., Nilssen E.A., Haugstad S.B., Angelsen B., Davies C.d.L. Ultrasound Mediated Delivery of Liposomal Doxorubicin in Prostate Tumor Tissue. Proceedings of the IEEE International Ultrasonics Symposium.

[54] Yudina A., Lepetit-Coiffé M., Moonen C.T.W. (2011). Evaluation of the Temporal Window for Drug Delivery Following Ultrasound-Mediated Membrane Permeability Enhancement. Mol. Imaging Biol..

[55] Larkin J.O., Casey G.D., Tangney M., Cashman J., Collins C.G., Soden D.M., O’Sullivan G.C. (2008). Effective Tumor Treatment Using Optimized Ultrasound-Mediated Delivery of Bleomycin. Ultrasound Med. Biol..

[56] Nelson J.L., Roeder B.L., Carmen J.C., Roloff F., Pitt W.G. (2002). Ultrasonically Activated Chemotherapeutic Drug Delivery in a Rat Model. Cancer Res..

[57] Rapoport N.Y., Kennedy A.M., Shea J.E., Scaife C.L., Nam K.-H. (2009). Controlled and Targeted Tumor Chemotherapy by Ultrasound-Activated Nanoemulsions/Microbubbles. J. Control. Release.

[58] Park E.-J., Zhang Y.-Z., Vykhodtseva N., McDannold N. (2012). Ultrasound-Mediated Blood-Brain/Blood-Tumor Barrier Disruption Improves Outcomes with Trastuzumab in a Breast Cancer Brain Metastasis Model. J. Control. Release.

[59] Wei K.-C., Chu P.-C., Wang H.-Y., Huang C.-Y., Chen P.-Y., Tsai H.-C., Lu Y.-J., Lee P.-Y., Tseng I.-C., Feng L.-Y. (2013). Focused Ultrasound-Induced Blood-Brain Barrier Opening to Enhance Temozolomide Delivery for Glioblastoma Treatment: A Preclinical Study. PLoS ONE.

[60] Kovacs Z., Werner B., Rassi A., Sass J.O., Martin-Fiori E., Bernasconi M. (2014). Prolonged Survival upon Ultrasound-Enhanced Doxorubicin Delivery in Two Syngenic Glioblastoma Mouse Models. J. Control. Release.

[61] McDannold N., Vykhodtseva N., Hynynen K. (2006). Targeted Disruption of the Blood–Brain Barrier with Focused Ultrasound: Association with Cavitation Activity. Phys. Med. Biol..

[62] Hynynen K., McDannold N., Vykhodtseva N., Jolesz F.A. (2001). Noninvasive MR Imaging–Guided Focal Opening of the Blood-Brain Barrier in Rabbits. Radiology.

[63] Treat L.H., McDannold N., Vykhodtseva N., Zhang Y., Tam K., Hynynen K. (2007). Targeted Delivery of Doxorubicin to the Rat Brain at Therapeutic Levels Using MRI-Guided Focused Ultrasound. Int. J. Cancer.

[64] Guthkelch A.N., Carter L.P., Cassady J.R., Hynynen K.H., Iacono R.P., Johnson P.C., Obbens E.A.M.T., Roemer R.B., Seeger J.F., Shimm D.S. (1991). Treatment of Malignant Brain Tumors with Focused Ultrasound Hyperthermia and Radiation: Results of a Phase I Trial. J. Neurooncol..

[65] Saito M., Mazda O., Takahashi K.A., Arai Y., Kishida T., Shin-Ya M., Inoue A., Tonomura H., Sakao K., Morihara T. (2007). Sonoporation Mediated Transduction of PDNA/SiRNA into Joint Synovium in Vivo. J. Orthop. Res..

[66] Kinoshita M., Hynynen K. (2005). A Novel Method for the Intracellular Delivery of SiRNA Using Microbubble-Enhanced Focused Ultrasound. Biochem. Biophys. Res. Commun..

[67] Taniyama Y., Tachibana K., Hiraoka K., Namba T., Yamasaki K., Hashiya N., Aoki M., Ogihara T., Yasufumi K., Morishita R. (2002). Local Delivery of Plasmid DNA Into Rat Carotid Artery Using Ultrasound. Circulation.

[68] Krasovitski B., Kimmel E. (2004). Shear Stress Induced by a Gas Bubble Pulsating in an Ultrasonic Field near a Wall. IEEE Trans. Ultrason. Ferroelectr. Freq. Control.

[69] Catania A.E., Ferrari A., Manno M., Spessa E. (2006). A Comprehensive Thermodynamic Approach to Acoustic Cavitation Simulation in High-Pressure Injection Systems by a Conservative Homogeneous Two-Phase Barotropic Flow Model. J. Eng. Gas Turbines Power.

[70] Bowden F.P., Brunton J.H. (1961). The Deformation of Solids by Liquid Impact at Supersonic Speeds. Proc. R. Soc. A Math. Phys. Eng. Sci..

[71] Pecha R., Wang Z.Q., Gompf B., Eisenmenger W. (1999). Single-bubble Sonoluminescence: Investigation of the Emitted Pressure Wave with a Streak Camera and a Fiber-optic Probe Hydrophone. J. Acoust. Soc. Am..

[72] Prosperetti A. (1997). A New Mechanism for Sonoluminescence. J. Acoust. Soc. Am..

[73] Brujan E.A., Nahen K., Schmidt P., Vogel A. (2001). Dynamics of Laser-Induced Cavitation Bubbles near an Elastic Boundary. J. Fluid Mech..

[74] Hajri Z., Boukadoum M., Hamam H., Fontaine R. (2005). An Investigation of the Physical Forces Leading to Thrombosis Disruption by Cavitation. J. Thromb. Thrombolysis.

[75] Kodama T., Takayama K. (1998). Dynamic Behavior of Bubbles During Extracorporeal. Ultrasound Med. Biol..

[76] Wörle K., Steinbach P., Hofstädter F. (1994). The Combined Effects of High-Energy Shock Waves and Cytostatic Drugs or Cytokines on Human Bladder Cancer Cells. Br. J. Cancer.

[77] Steinbach P., Hofstädter F., Nicolai H., Rössler W., Wieland W. (1992). In Vitro Investigations on Cellular Damage Induced by High Energy Shock Waves. Ultrasound Med. Biol..

[78] Couture O., Foley J., Kassell N., Larrat B., Aubry J.-F. (2014). Review of Ultrasound Mediated Drug Delivery for Cancer Treatment: Updates from Pre-Clinical Studies. Transl. Cancer Res..

[79] Geers B., Dewitte H., De Smedt S.C., Lentacker I. (2012). Crucial Factors and Emerging Concepts in Ultrasound-Triggered Drug Delivery. J. Control. Release.

[80] Enden G., Schroeder A. (2009). A Mathematical Model of Drug Release from Liposomes by Low Frequency Ultrasound. Ann. Biomed. Eng..

[81] Schroeder A., Avnir Y., Weisman S., Najajreh Y., Gabizon A., Talmon Y., Kost J., Barenholz Y. (2007). Controlling Liposomal Drug Release with Low Frequency Ultrasound: Mechanism and Feasibility. Langmuir.

[82] Afadzi M., Davies C.d.L., Hansen Y.H., Johansen T., Standal Ø.K., Hansen R., Måsøy S.E., Nilssen E.A., Angelsen B. (2012). Effect of Ultrasound Parameters on the Release of Liposomal Calcein. Ultrasound Med. Biol..

[83] Lin Y., Lin L., Cheng M., Jin L., Du L., Han T., Xu L., Yu A.C.H., Qin P. (2016). Effect of Acoustic Parameters on the Cavitation Behavior of SonoVue Microbubbles Induced by Pulsed Ultrasound. Ultrason. Sonochem..

[84] Mannaris C., Averkiou M.A. (2012). Investigation of Microbubble Response to Long Pulses Used in Ultrasound-Enhanced Drug Delivery. Ultrasound Med. Biol..

[85] Mayer R., Schenk E., Child S., Norton S., Cox C., Hartman C., Cox C., Carstensen E. (1990). Pressure Threshold for Shock Wave Induced Renal Hemorrhage. J. Urol..

[86] Child S.Z., Hartman C.L., Schery L.A., Carstensen E.L. (1990). Lung Damage from Exposure to Pulsed Ultrasound. Ultrasound Med. Biol..

[87] Coussios C.C., Farny C.H., ter Haar G., Roy R.A. (2007). Role of Acoustic Cavitation in the Delivery and Monitoring of Cancer Treatment by High-Intensity Focused Ultrasound (HIFU). Int. J. Hyperth..

[88] Kennedy J.E. (2005). High-Intensity Focused Ultrasound in the Treatment of Solid Tumours. Nat. Rev. Cancer.

[89] Hancock H., Dreher M.R., Crawford N., Pollock C.B., Shih J., Wood B.J., Hunter K., Frenkel V. (2009). Evaluation of Pulsed High Intensity Focused Ultrasound Exposures on Metastasis in a Murine Model. Clin. Exp. Metastasis.

[90] Steinbach P., Hofstaedter F., Nicolai H., Roessler W., Wieland W. (1993). Determination of the Energy-Dependent Extent of Vascular Damage Caused by High-Energy Shock Waves in an Umbilical Cord Model. Urol. Res..

[91] Needham D., Anyarambhatla G., Kong G., Dewhirst M.W. (2000). A New Temperature-Sensitive Liposome for Use with Mild Hyperthermia: Characterization and Testing in a Human Tumor Xenograft Model. Cancer Res..

[92] Huang S.L., MacDonald R.C. (2004). Acoustically Active Liposomes for Drug Encapsulation and Ultrasound-Triggered Release. Biochim. Biophys. Acta Biomembr..

[93] Lin H.Y., Thomas J.L. (2003). PEG-Lipids and Oligo (Ethylene Glycol) Surfactants Enhance the Ultrasonic Permeabilizability of Liposomes. Langmuir.

[94] Lin H.Y., Thomas J.L. Pluronic P105 Sensitizes Cholesterol-Free Liposomes to Ultrasound. Proceedings of the the IEEE Annual Northeast Bioengineering Conference.

[95] Lin H.Y., Thomas J.L. (2004). Factors Affecting Responsivity of Unilamellar Liposomes to 20 KHz Ultrasound. Langmuir.

[96] Tirosh O., Barenholz Y., Katzhendler J., Priev A. (1998). Hydration of Polyethylene Glycol-Grafted Liposomes. Biophys. J..

[97] Evjen T.J., Nilssen E.A., Barnert S., Schubert R., Brandl M., Fossheim S.L. (2011). Ultrasound-Mediated Destabilization and Drug Release from Liposomes Comprising Dioleoylphosphatidylethanolamine. Eur. J. Pharm. Sci..

[98] Tata D.B., Dunn F. (1992). Ultrasound and Model Membrane Systems: Analyses and Predlctions. J. Phys. Chem..

[99] Maynard V.M., Magin R.L., Storm-Jensen P.R., Dunn F. (1983). Ultrasonic Absorption by Liposomes. Ultrason. Symp..

[100] Wu F., Wang Z.-B., Chen W.-Z., Zou J.-Z., Bai J., Zhu H., Li K.-Q., Jin C.-B., Xie F.-L., Su H.-B. (2005). Advanced Hepatocellular Carcinoma: Treatment with High-Intensity Focused Ultrasound Ablation Combined with Transcatheter Arterial Embolization. Radiology.

[101] Wu F., Wang Z.-B., Jin C.-B., Zhang J.-P., Chen W.-Z., Bai J., Zou J.-Z., Zhu H. (2004). Circulating Tumor Cells in Patients with Solid Malignancy Treated by High-Intensity Focused Ultrasound. Ultrasound Med. Biol..

[102] Mafune K., Tanaka Y. (2000). Influence of Multimodality Therapy on the Cellular Immunity of Patients with Esophageal Cancer. Ann. Surg. Oncol..

[103] Wu F., Wang Z.-B., Lu P., Xu Z.-L., Chen W.-Z., Zhu H., Jin C.-B. (2004). Activated Anti-Tumor Immunity in Cancer Patients after High Intensity Focused Ultrasound Ablation. Ultrasound Med. Biol..

[104] Kramer G., Steiner G.E., Gröbl M., Hrachowitz K., Reithmayr F., Paucz L., Newman M., Madersbacher S., Gruber D., Susani M. (2004). Response to Sublethal Heat Treatment of Prostatic Tumor Cells and of Prostatic Tumor Infiltrating T-Cells. Prostate.

[105] Schueller G., Kettenbach J., Sedivy R., Bergmeister H., Stift A., Fried J., Gnant M., Lammer J. (2004). Expression of Heat Shock Proteins in Human Hepatocellular Carcinoma after Radiofrequency Ablation in an Animal Model. Oncol. Rep..

[106] Den Brok M.H.M.G.M., Sutmuller R.P.M., van der Voort R., Bennink E.J., Figdor C.G., Ruers T.J.M., Adema G.J. (2004). In Situ Tumor Ablation Creates an Antigen Source for the Generation of Antitumor Immunity. Cancer Res..

[107] Leslie T., Ritchie R., Illing R., Ter Haar G., Phillips R., Middleton M., Bch B., Wu F., Cranston D. (2012). High-Intensity Focused Ultrasound Treatment of Liver Tumours: Post-Treatment MRI Correlates Well with Intra-Operative Estimates of Treatment Volume. Br. J. Radiol..

[108] Wu F., Wang Z.-B., Chen W.-Z., Zou J.-Z., Bai J., Zhu H., Li K.-Q., Xie F.-L., Jin C.-B., Su H.-B. (2004). Extracorporeal Focused Ultrasound Surgery for Treatment of Human Solid Carcinomas: Early Chinese Clinical Experience. Ultrasound Med. Biol..

[109] Visioli A., Rivens I., ter Haar G., Horwich A., Huddart R., Moskovic E., Padhani A., Glees J. (1999). Preliminary Results of a Phase I Dose Escalation Clinical Trial Using Focused Ultrasound in the Treatment of Localised Tumours. Eur. J. Ultrasound.

[110] Vallancien G., Harouni M., Guillonneau B., Veillon B., Bougaran J. (1996). Ablation of Superficial Bladder Tumors with Focused Extracorporeal Pyrotherapy. Urology.

[111] Zagar T.M., Vujaskovic Z., Formenti S., Rugo H., Muggia F., O’Connor B., Myerson R., Stauffer P., Hsu I.C., Diederich C. (2014). Two Phase I Dose-Escalation/Pharmacokinetics Studies of Low Temperature Liposomal Doxorubicin (LTLD) and Mild Local Hyperthermia in Heavily Pretreated Patients with Local Regionally Recurrent Breast Cancer. Int. J. Hyperth..

[112] Gray M.D., Lyon P.C., Mannaris D.C., Folkes L.K., Stratford M., Campo L., Chung D.D.Y.F., Scott F.S., Gleeson F.V., Coussios F.C.C. (2019). Focused Ultrasound Hyperthermia for Targeted Drug Release from Thermosensitive Liposomes: Results from a Phase I Trial. Radiology.

[113] Lyon P.C., Gray M.D., Mannaris C., Folkes L.K., Stratford M., Campo L., Chung D.Y.F., Scott S., Anderson M., Goldin R. (2018). Safety and Feasibility of Ultrasound-Triggered Targeted Drug Delivery of Doxorubicin from Thermosensitive Liposomes in Liver Tumours (TARDOX): A Single-Centre, Open-Label, Phase 1 Trial. Lancet Oncol..

[114] Dromi S., Frenkel V., Luk A., Traughber B., Angstadt M., Bur M., Poff J., Xie J., Libutti S.K., Li K.C.P. (2007). Pulsed-High Intensity Focused Ultrasound and Low Temperature—Sensitive Liposomes for Enhanced Targeted Drug Delivery and Antitumor Effect. Clin. Cancer Res..

[115] De Smet M., Heijman E., Langereis S., Hijnen N.M., Grüll H. (2011). Magnetic Resonance Imaging of High Intensity Focused Ultrasound Mediated Drug Delivery from Temperature-Sensitive Liposomes: An in Vivo Proof-of-Concept Study. J. Control. Release.

[116] Negussie A.H., Yarmolenko P.S., Partanen A., Ranjan A., Jacobs G., Woods D., Bryant H., Thomasson D., Dewhirst M.W., Wood B.J. (2011). Formulation and Characterisation of Magnetic Resonance Imageable Thermally Sensitive Liposomes for Use with Magnetic Resonance-Guided High Intensity Focused Ultrasound. Int. J. Hyperth..

[117] Ranjan A., Jacobs G.C., Woods D.L., Negussie A.H., Partanen A., Yarmolenko P.S., Gacchina C.E., Sharma K.V., Frenkel V., Wood B.J. (2012). Image-Guided Drug Delivery with Magnetic Resonance Guided High Intensity Focused Ultrasound and Temperature Sensitive Liposomes in a Rabbit Vx2 Tumor Model. J. Control. Release.

[118] Schroeder A., Honen R., Turjeman K., Gabizon A., Kost J., Barenholz Y. (2009). Ultrasound Triggered Release of Cisplatin from Liposomes in Murine Tumors. J. Control. Release.

[119] Li Y., An H., Wang X., Wang P., Qu F., Jiao Y., Zhang K., Liu Q. (2018). Ultrasound-Triggered Release of Sinoporphyrin Sodium from Liposome-Microbubble Complexes and Its Enhanced Sonodynamic Toxicity in Breast Cancer. Nano Res..

[120] Hagtvet E., Evjen T.J., Olsen D.R., Fossheim S.L., Nilssen E.A. (2011). Ultrasound Enhanced Antitumor Activity of Liposomal Doxorubicin in Mice. J. Drug Target..

[121] Aryal M., Vykhodtseva N., Zhang Y.-Z., Park J., McDannold N. (2013). Multiple Treatments with Liposomal Doxorubicin and Ultrasound-Induced Disruption of Blood-Tumor and Blood-Brain Barriers Improve Outcomes in a Rat Glioma Model. J. Control. Release.

[122] Rugo H., Pabbathi H., Shrestha S., Aithal S., Borys N., Musso L., Zoberi I. Lyso-Thermosensitive Liposomal Doxorubicin shows efficacy with minimal adverse events in patients with breast cancer recurrence at the chest wall. Proceedings of the the San Antonio Breast Cancer Symposium.

[123] Delfino J.G., Woods T.O. (2016). New Developments in Standards for MRI Safety Testing of Medical Devices. Curr. Radiol. Rep..

[124] Noebauer-Huhmann I.M., Weber M.A., Lalam R.K., Trattnig S., Bohndorf K., Vanhoenacker F., Tagliafico A., Van Rijswijk C., Vilanova J.C., Afonso P.D. (2015). Erratum: Soft Tissue Tumors in Adults: ESSR-Approved Guidelines for Diagnostic Imaging. Semin. Musculoskelet. Radiol..

[125] Sanz B., Calatayud M.P., Torres T.E., Fanarraga M.L., Ibarra M.R., Goya G.F. (2017). Magnetic Hyperthermia Enhances Cell Toxicity with Respect to Exogenous Heating. Biomaterials.

[126] Kozissnik B., Bohorquez A.C., Dobson J., Rinaldi C. (2013). Magnetic Fluid Hyperthermia: Advances, Challenges, and Opportunity. Int. J. Hyperth..

[127] Maier-Hauff K., Ulrich F., Nestler D., Orawa H., Budach V., Jordan A. (2011). Efficacy and Safety of Intratumoral Thermotherapy Using Magnetic Iron-Oxide Nanoparticles Combined with External Beam Radiotherapy on Patients with Recurrent Glioblastoma Multiforme. J. Neurooncol..

[128] Mura S., Nicolas J., Couvreur P. (2013). Stimuli-Responsive Nanocarriers for Drug Delivery. Nat. Mater..

[129] Hilger I., Kaiser W.A. (2012). Iron Oxide-Based Nanostructures for MRI and Magnetic Hyperthermia. Nanomedicine.

[130] Bonini M., Berti D., Baglioni P. (2013). Current Opinion in Colloid & Interface Science Nanostructures for Magnetically Triggered Release of Drugs and Biomolecules. Curr. Opin. Colloid Interface Sci..

[131] Gautier J., Munnier E., Soucé M., Chourpa I. (2013). Recent Advances in Theranostic Nanocarriers of Doxorubicin Based on Iron Oxide and Gold Nanoparticles. J. Control. Release.

[132] Clares B., Biedma-ortiz R.A., Sáez-fernández E., Prados J.C., Melguizo C., Cabeza L., Ortiz R., Arias J.L. (2013). Nano-Engineering of 5-Fluorouracil-Loaded Magnetoliposomes for Combined Hyperthermia and Chemotherapy against Colon Cancer. Eur. J. Pharm. Biopharm..

[133] Lee J.H., Chen K.J., Noh S.H., Garcia M.A., Wang H., Lin W.Y., Jeong H., Kong B.J., Stout D.B., Cheon J. (2013). On-Demand Drug Release System for In Vivo Cancer Treatment through Self-Assembled Magnetic Nanoparticles. Angew. Chem. Int. Ed. Engl..

[134] Hayashi K., Nakamura M., Miki H., Ozaki S., Abe M. (2014). Magnetically Responsive Smart Nanoparticles for Cancer Treatment with a Combination of Magnetic Hyperthermia and Remote-Control Drug Release. Theranostics.

[135] Deok S., Sartor M., Hu C.J., Zhang W., Zhang L., Jin S. (2013). Magnetic Field Activated Lipid–Polymer Hybrid Nanoparticles for Stimuli-Responsive Drug Release. Acta Biomater..

[136] Hayashi K., Ono K., Suzuki H., Sawada M., Moriya M. (2010). Drug Release from Magnetic Nanoparticle/Organic Hybrid Based on Hyperthermic Effect. ACS Appl. Mater. Interfaces.

[137] Chiang W., Ke C., Liao Z., Chen S., Chen F. (2012). Pulsatile Drug Release from PLGA Hollow Microspheres by Controlling the Permeability of Their Walls with a Magnetic Field. Small.

[138] Dou Y., Hynynen K., Allen C. (2017). To Heat or Not to Heat: Challenges with Clinical Translation of Thermosensitive Liposomes. J. Control. Release.

[139] Guo Y., Zhang Y., Ma J., Li Q., Li Y., Zhou X., Zhao D., Song H., Chen Q., Zhu X. (2018). Light/Magnetic Hyperthermia Triggered Drug Released from Multi-Functional Thermo-Sensitive Magnetoliposomes for Precise Cancer Synergetic Theranostics. J. Control. Release.

[140] Gogoi M., Jaiswal M.K., Sarma H.D., Bahadur D., Banerjee R. (2017). Biocompatibility and Therapeutic Evaluation of Magnetic Liposomes Designed for Self-Controlled Cancer Hyperthermia and Chemotherapy. Integr. Biol. (U. K.).

[141] Wang L., Zhang J., An Y., Wang Z., Liu J., Li Y., Zhang D. (2011). A Study on the Thermochemotherapy Effect of Nanosized As_2_O_3_/MZF Thermosensitive Magnetoliposomes on Experimental Hepatoma in Vitro and in Vivo. Nanotechnology.

[142] Nappini S., Fogli S., Castroflorio B., Bonini M., Baldelli Bombelli F., Baglioni P. (2016). Magnetic Field Responsive Drug Release from Magnetoliposomes in Biological Fluids. J. Mater. Chem. B.

[143] Amstad E., Kohlbrecher J., Müller E., Schweizer T., Textor M., Reimhult E. (2011). Triggered Release from Liposomes through Magnetic Actuation of Iron Oxide Nanoparticle Containing Membranes. Nano Lett..

[144] Tai L.A., Tsai P.J., Wang Y.C., Wang Y.J., Lo L.W., Yang C.S. (2009). Thermosensitive Liposomes Entrapping Iron Oxide Nanoparticles for Controllable Drug Release. Nanotechnology.

[145] Kim D.H., Rozhkova E.A., Ulasov I.V., Bader S.D., Rajh T., Lesniak M.S., Novosad V. (2010). Biofunctionalized Magnetic-Vortex Microdiscs for Targeted Cancer-Cell Destruction. Nat. Mater..

[146] Joniec A., Sek S., Krysinski P. (2016). Magnetoliposomes as Potential Carriers of Doxorubicin to Tumours. Chem. A Eur. J..

[147] Zhang E., Kircher M.F., Koch M., Eliasson L., Goldberg S.N., Renström E. (2014). Dynamic Magnetic Fields Remote-Control Apoptosis via Nanoparticle Rotation. ACS Nano.

[148] Podaru G., Ogden S., Baxter A., Shrestha T., Ren S., Thapa P., Dani R.K., Wang H., Basel M.T., Prakash P. (2014). Pulsed Magnetic Field Induced Fast Drug Release from Magneto Liposomes via Ultrasound Generation. J. Phys. Chem. B.

[149] Reddy L.H., Arias J.L., Nicolas J., Couvreur P. (2012). Magnetic Nanoparticles: Design and Characterization, Toxicity and Biocompatibility, Pharmaceutical and Biomedical Applications. Chem. Rev..

[150] Deatsch A.E., Evans B.A. (2014). Heating Efficiency in Magnetic Nanoparticle Hyperthermia. J. Magn. Magn. Mater..

[151] Rosensweig R.E. (2002). Heating Magnetic Fluid with Alternating Magnetic Field. J. Magn. Magn. Mater..

[152] Carrey J., Mehdaoui B., Respaud M. (2015). Simple Models for Dynamic Hysteresis Loop Calculations of Magnetic Single-Domain Nanoparticles: Application to Magnetic Hyperthermia Optimization. J. Appl. Phys..

[153] Riedinger A., Guardia P., Curcio A., Garcia M.A., Cingolani R., Manna L., Pellegrino T. (2013). Subnanometer Local Temperature Probing and Remotely Controlled Drug Release Based on Azo-Functionalized Iron Oxide Nanoparticles. Nano Lett..

[154] Dutz S., Hergt R. (2013). Magnetic Nanoparticle Heating and Heat Transfer on a Microscale: Basic Principles, Realities and Physical Limitations of Hyperthermia for Tumour Therapy. Int. J. Hyperth..

[155] Cheng Y., Muroski M.E., Petit D.C.M.C., Mansell R., Vemulkar T., Morshed R.A., Han Y., Balyasnikova I.V., Horbinski C.M., Huang X. (2016). Rotating Magnetic Field Induced Oscillation of Magnetic Particles for in Vivo Mechanical Destruction of Malignant Glioma. J. Control. Release.

[156] Zhang L., Zhao Y., Wang X. (2017). Nanoparticle-Mediated Mechanical Destruction of Cell Membranes: A Coarse-Grained Molecular Dynamics Study. ACS Appl. Mater. Interfaces.

[157] Cheng D., Li X., Zhang G., Shi H. (2014). Morphological Effect of Oscillating Magnetic Nanoparticles in Killing Tumor Cells. Nanoscale Res. Lett..

[158] Rikken R.S.M., Nolte R.J.M., Maan J.C., Van Hest J.C.M., Wilson D.A., Christianen P.C.M. (2014). Manipulation of Micro- and Nanostructure Motion with Magnetic Fields. Soft Matter.

[159] Ortega D., Pankhurst Q.A. (2013). Magnetic Hyperthermia. Nanoscience.

[160] Pradhan P., Giri J., Rieken F., Koch C., Mykhaylyk O., Döblinger M., Banerjee R., Bahadur D., Plank C. (2010). Targeted Temperature Sensitive Magnetic Liposomes for Thermo-Chemotherapy. J. Control. Release.

[161] Peller M., Willerding L., Limmer S., Hossann M., Dietrich O., Ingrisch M., Sroka R., Lindner L.H. (2016). Surrogate MRI Markers for Hyperthermia-Induced Release of Doxorubicin from Thermosensitive Liposomes in Tumors. J. Control. Release.

[162] Lu A.H., Salabas E.L., Schüth F. (2007). Magnetic Nanoparticles: Synthesis, Protection, Functionalization, and Application. Angew. Chem. Int. Ed..

[163] Hedayatnasab Z., Abnisa F., Daud W.M.A.W. (2017). Review on Magnetic Nanoparticles for Magnetic Nanofluid Hyperthermia Application. Mater. Des..

[164] Das R., Alonso J., Nemati Porshokouh Z., Kalappattil V., Torres D., Phan M.H., Garaio E., García J.Á., Sanchez Llamazares J.L., Srikanth H. (2016). Tunable High Aspect Ratio Iron Oxide Nanorods for Enhanced Hyperthermia. J. Phys. Chem. C.

[165] Simeonidis K., Morales M.P., Marciello M., Angelakeris M., de la Presa P., Lazaro-Carrillo A., Tabero A., Villannueva A., Chubykalo O., Serantes D. (2016). In-Situ Particles Reorientation during Magnetic Hyperthermia Application: Shape Matters Twice. Sci. Rep..

[166] Abenojar E.C., Wickramasinghe S., Bas-Concepcion J., Samia A.C.S. (2016). Structural Effects on the Magnetic Hyperthermia Properties of Iron Oxide Nanoparticles. Prog. Nat. Sci. Mater. Int..

[167] Nemati Z., Alonso J., Martinez L.M., Khurshid H., Garaio E., Garcia J.A., Phan M.H., Srikanth H. (2016). Enhanced Magnetic Hyperthermia in Iron Oxide Nano-Octopods: Size and Anisotropy Effects. J. Phys. Chem. C.

[168] Espinosa A., Kolosnjaj-Tabi J., Abou-Hassan A., Plan Sangnier A., Curcio A., Silva A.K.A., Di Corato R., Neveu S., Pellegrino T., Liz-Marzán L.M. (2018). Magnetic (Hyper)Thermia or Photothermia? Progressive Comparison of Iron Oxide and Gold Nanoparticles Heating in Water, in Cells, and In Vivo. Adv. Funct. Mater..

[169] Walter A., Billotey C., Garofalo A., Ulhaq-Bouillet C., Lefevre C., Taleb J., Laurent S., Elst L.V., Uller R., Lartigue L. (2014). Mastering the Shape and Composition of Dendronized Iron Oxide Nanoparticles to Tailor Magnetic Resonance Imaging and Hyperthermia. Chem. Mater..

[170] Shen Y., Wu C., Uyeda T.Q.P., Plaza G.R., Liu B., Han Y., Lesniak M.S., Cheng Y. (2017). Elongated Nanoparticle Aggregates in Cancer Cells for Mechanical Destruction with Low Frequency Rotating Magnetic Field. Theranostics.

[171] Tietze R., Zaloga J., Unterweger H., Lyer S., Friedrich R.P., Janko C., Pöttler M., Dürr S., Alexiou C. (2015). Magnetic Nanoparticle-Based Drug Delivery for Cancer Therapy. Biochem. Biophys. Res. Commun..

[172] Dai M., Wu C., Wang X., Fang H.-M., Li L., Yan J.B., Zeng D.L., Zou T. (2017). Thermo-Responsive Magnetic Liposomes for Hyperthermia-Triggered Local Drug Delivery. J. Control. Release.

[173] García-Jimeno S., Escribano E., Queralt J., Estelrich J. (2012). External Magnetic Field-Induced Selective Biodistribution of Magnetoliposomes in Mice. Nanoscale Res. Lett..

[174] Delaney G.P., Barton M.B. (2015). Evidence-Based Estimates of the Demand for Radiotherapy. Clin. Oncol..

[175] Hainfeld J.F., Dilmanian F.A., Slatkin D.N., Smilowitz H.M. (2008). Radiotherapy Enhancement with Gold Nanoparticles. J. Pharm. Pharmacol..

[176] Schulz-Ertner D., Tsujii H. (2007). Particle Radiation Therapy Using Proton and Heavier Ion Beams. J. Clin. Oncol..

[177] Haume K., Rosa S., Grellet S., Śmiałek M.A., Butterworth K.T., Solov’yov A.V., Prise K.M., Golding J., Mason N.J. (2016). Gold Nanoparticles for Cancer Radiotherapy: A Review. Cancer Nanotechnol..

[178] Lo Nigro C., Denaro N., Merlotti A., Merlano M. (2017). Head and neck cancer: improving outcomes with a multidisciplinary approach. Cancer Manag. Res..

[179] Carlsson L., Bratman S.V., Siu L.L., Spreafico A. (2017). The Cisplatin Total Dose and Concomitant Radiation in Locoregionally Advanced Head and Neck Cancer: Any Recent Evidence for Dose Efficacy?. Curr. Treat. Options Oncol..

[180] Fayette J., Molin Y., Lavergne E., Montbarbon X., Racadot S., Poupart M., Ramade A., Zrounba P., Ceruse P., Pommier P. (2015). Radiotherapy potentiation with weekly cisplatin compared to standard every 3 weeks cisplatin chemotherapy for locoregionally advanced head and neck squamous cell carcinoma. Drug Des. Dev. Ther..

[181] Chang H.-I., Yeh M.-K. (2012). Clinical Development of Liposome-Based Drugs: Formulation, Characterization, and Therapeutic Efficacy. Int. J. Nanomed..

[182] Deng W., Chen W., Clement S., Guller A., Zhao Z., Engel A., Goldys E.M. (2018). Controlled Gene and Drug Release from a Liposomal Delivery Platform Triggered by X-Ray Radiation. Nat. Commun..

[183] Gutteridge J.M.C., Halliwell B. (1990). The Measurement and Mechanism of Lipid Peroxidation in Biological Systems. Trends Biochem. Sci..

[184] Sicard-Roselli C., Brun E., Gilles M., Baldacchino G., Kelsey C., McQuaid H., Polin C., Wardlow N., Currell F. (2014). A New Mechanism for Hydroxyl Radical Production in Irradiated Nanoparticle Solutions. Small.

[185] Ashton J.R., Castle K.D., Qi Y., Kirsch D.G., West J.L., Badea C.T. (2018). Dual-Energy CT Imaging of Tumor Liposome Delivery after Gold Nanoparticle-Augmented Radiation Therapy. Theranostics.

[186] Schuemann J., Berbeco R., Chithrani D.B., Cho S.H., Kumar R., McMahon S.J., Sridhar S., Krishnan S. (2016). Roadmap to Clinical Use of Gold Nanoparticles for Radiation Sensitization. Int. J. Radiat. Oncol. Biol. Phys..

[187] Hainfeld J.F., Slatkin D.N., Smilowitz H.M. (2004). The Use of Gold Nanoparticles to Enhance Radiotherapy in Mice. Phys. Med. Biol..

[188] Cho S.H. (2005). Estimation of Tumour Dose Enhancement Due to Gold Nanoparticles During Typical Radiation Treatments: A Preliminary Monte Carlo Study. Phys. Med. Biol..

[189] Her S., Jaffray D.A., Allen C. (2017). Gold Nanoparticles for Applications in Cancer Radiotherapy: Mechanisms and Recent Advancements. Adv. Drug Deliv. Rev..

[190] Brun E., Sanche L., Sicard-Roselli C. (2009). Parameters Governing Gold Nanoparticle X-Ray Radiosensitization of DNA in Solution. Colloids Surf. B Biointerfaces.

[191] Lechtman E., Chattopadhyay N., Cai Z., Mashouf S., Reilly R., Pignol J.P. (2011). Implications on Clinical Scenario of Gold Nanoparticle Radiosensitization in Regards to Photon Energy, Nanoparticle Size, Concentration and Location. Phys. Med. Biol..

[192] Leung M.K.K., Chow J.C.L., Chithrani B.D., Lee M.J.G., Oms B., Jaffray D.A. (2011). Irradiation of Gold Nanoparticles by X-Rays: Monte Carlo Simulation of Dose Enhancements and the Spatial Properties of the Secondary Electrons Production. Med. Phys..

[193] Jones B.L., Krishnan S., Cho S.H. (2010). Estimation of Microscopic Dose Enhancement Factor around Gold Nanoparticles by Monte Carlo Calculations. Med. Phys..

[194] Hwang C., Kim J.M., Kim J. (2017). Influence of Concentration, Nanoparticle Size, Beam Energy, and Material on Dose Enhancement in Radiation Therapy. J. Radiat. Res..

[195] Butterworth K.T., Mcmahon S.J., Taggart L.E., Prise K.M. (2013). Radiosensitization by Gold Nanoparticles: Effective at Megavoltage Energies and Potential Role of Oxidative Stress. Transl. Cancer Res..

[196] Chang M.Y., Shiau A.L., Chen Y.H., Chang C.J., Chen H.H.W., Wu C.L. (2008). Increased Apoptotic Potential and Dose-Enhancing Effect of Gold Nanoparticles in Combination with Single-Dose Clinical Electron Beams on Tumor-Bearing Mice. Cancer Sci..

[197] Yang C., Bromma K., Sung W., Schuemann J., Chithrani D. (2018). Determining the Radiation Enhancement Effects of Gold Nanoparticles in Cells in a Combined Treatment with Cisplatin and Radiation at Therapeutic Megavoltage Energies. Cancers.

[198] Rahman W.N., Bishara N., Ackerly T., He C.F., Jackson P., Wong C., Davidson R., Geso M. (2009). Enhancement of Radiation Effects by Gold Nanoparticles for Superficial Radiation Therapy. Nanomed. Nanotechnol. Biol. Med..

[199] Schardt D., Elsässer T., Schulz-Ertner D. (2010). Heavy-Ion Tumor Therapy: Physical and Radiobiological Benefits. Rev. Mod. Phys..

[200] Verkhovtsev A.V., Korol A.V., Solov’Yov A.V. (2015). Revealing the Mechanism of the Low-Energy Electron Yield Enhancement from Sensitizing Nanoparticles. Phys. Rev. Lett..

[201] Lin Y., McMahon S.J., Paganetti H., Schuemann J. (2015). Biological Modeling of Gold Nanoparticle Enhanced Radiotherapy for Proton Therapy. Phys. Med. Biol..

[202] Lukianova-Helb E.Y., Ren X., Sawant R.R., Wu X., Torchilin V.P., Lapotko D.O. (2014). On-demand intracellular amplification of chemoradiation with cancer-specific plasmonic nanobubbles. Nat. Med..

[203] Davies M.J. (2016). Protein Oxidation and Peroxidation. Biochem. J..

[204] Park S.H., Oh S.G., Mun J.Y., Han S.S. (2006). Loading of Gold Nanoparticles inside the DPPC Bilayers of Liposome and Their Effects on Membrane Fluidities. Colloids Surf. B Biointerfaces.

[205] Rengan A.K., Bukhari A.B., Pradhan A., Malhotra R., Banerjee R., Srivastava R., De A. (2015). In Vivo Analysis of Biodegradable Liposome Gold Nanoparticles as Efficient Agents for Photothermal Therapy of Cancer. Nano Lett..

[206] Hobbs S.K., Monsky W.L., Yuan F., Roberts W.G., Griffith L., Torchilin V.P., Jain R.K. (1998). Regulation of Transport Pathways in Tumor Vessels: Role of Tumor Type and Microenvironment. Proc. Natl. Acad. Sci. USA.

[207] Wicki A., Witzigmann D., Balasubramanian V., Huwyler J. (2015). Nanomedicine in Cancer Therapy: Challenges, Opportunities, and Clinical Applications. J. Control. Release.

[208] Lammers T., Kiessling F., Hennink W.E., Storm G. (2012). Drug targeting to tumors: Principles, pitfalls and (pre-) clinical progress. J. Control. Release.

[209] Dreaden E.C., Alkilany A.M., Huang X., Murphy C.J., El-Sayed M.A. (2012). The Golden Age: Gold Nanoparticles for Biomedicine. Chem. Soc. Rev..

[210] Chen Y.S., Hung Y.C., Liau I., Huang G.S. (2009). Assessment of the in Vivo Toxicity of Gold Nanoparticles. Nanoscale Res. Lett..

[211] Lasagna-Reeves C., Gonzalez-Romero D., Barria M.A., Olmedo I., Clos A., Sadagopa Ramanujam V.M., Urayama A., Vergara L., Kogan M.J., Soto C. (2010). Bioaccumulation and Toxicity of Gold Nanoparticles after Repeated Administration in Mice. Biochem. Biophys. Res. Commun..

[212] Libutti S.K., Paciotti G.F., Byrnes A.A., Alexander H.R., Gannon W.E., Walker M., Seidel G.D., Yuldasheva N., Tamarkin L. (2010). Phase I and Pharmacokinetic Studies of CYT-6091, a Novel PEGylated Colloidal Gold-RhTNF Nanomedicine. Clin. Cancer Res..

[213] Singh P., Pandit S., Mokkapati V.R.S.S., Garg A., Ravikumar V., Mijakovic I. (2018). Gold Nanoparticles in Diagnostics and Therapeutics for Human Cancer. Int. J. Mol. Sci..

[214] Jeene P.M., van Laarhoven H.W.M., Hulshof M.C.C.M. (2018). The Role of Definitive Chemoradiation in Patients with Non-Metastatic Oesophageal Cancer. Best Pract. Res. Clin. Gastroenterol..

[215] Faivre-Finn C., Snee M., Ashcroft L., Appel W., Barlesi F., Bhatnagar A., Bezjak A., Cardenal F., Fournel P., Harden S. (2017). Concurrent Once-Daily versus Twice-Daily Chemoradiotherapy in Patients with Limited-Stage Small-Cell Lung Cancer (CONVERT): An Open-Label, Phase 3, Randomised, Superiority Trial. Lancet Oncol..

[216] Gadducci A., Barsotti C., Laliscia C., Cosio S., Fanucchi A., Tana R., Fabrini M.G. (2017). Dose-Dense Paclitaxel- and Carboplatin-Based Neoadjuvant Chemotherapy Followed by Surgery or Concurrent Chemoradiotherapy in Cervical Cancer: A Preliminary Analysis. Anticancer Res..

[217] Hoskin P., Bloomfield D., Dickson J., Jena R., Misra V., Prestwich R. (2016). Radiotherapy Dose Fractionation. Clin. Oncol..

[218] Kamkaew A., Chen F., Zhan Y., Majewski R.L., Cai W. (2016). Scintillating Nanoparticles as Energy Mediators for Enhanced Photodynamic Therapy. ACS Nano.

